# Synthesis of New 1, 3, 4-Oxadiazole-Incorporated 1, 2, 3-Triazole Moieties as Potential Anticancer Agents Targeting Thymidylate Synthase and Their Docking Studies

**DOI:** 10.3390/ph13110390

**Published:** 2020-11-14

**Authors:** Mohammad Mahboob Alam, Abdulraheem SA Almalki, Thikryat Neamatallah, Nada M. Ali, Azizah M. Malebari, Syed Nazreen

**Affiliations:** 1 Department of Chemistry, Faculty of Science, Albaha University, Albaha-1988, Saudi Arabia; mmalamchem@gmail.com (M.M.A.); umyousee@gmail.com (N.M.A.); 2 Department of Chemistry, Faculty of Science, Taif University, Taif-21974, Saudi Arabia; drasaalmalki1@gmail.com; 3 Department of Pharmacology and Toxicology, Faculty of Pharmacy, King Abdulaziz University, Jeddah 21589, Saudi Arabia; Taneamatallah@kau.edu.sa; 4 Department of Pharmaceutical Chemistry, Faculty of Pharmacy, King Abdulaziz University, Jeddah 21589, Saudi Arabia; amelibary@kau.edu.sa

**Keywords:** thymidylate synthase, cytotoxicity, 1,2,3-triazole, 1,3,4-oxadiazole, 5-fluoruracil, pemetrexed, docking

## Abstract

Thymidylate synthase (TS) has emerged as a hot spot in cancer treatment, as it is directly involved in DNA synthesis. In the present article, nine hybrids containing 1,2,3-triazole and 1,3,4-oxadiazole moieties (**6**–**14**) were synthesized and evaluated for anticancer and in vitro thymidylate synthase activities. According to in silico pharmacokinetic studies, the synthesized hybrids exhibited good drug likeness properties and bioavailability. The cytotoxicity results indicated that compounds **12** and **13** exhibited remarkable inhibition on the tested Michigan Cancer Foundation (MCF-7) and Human colorectal Carcinoma (HCT-116) cell lines. Compound **12** showed four-fold inhibition to a standard drug, 5-fluoruracil, and comparable inhibition to tamoxifen, whereas compound **13** exerted five-fold activity of tamoxifen and 24-fold activity of 5-fluorouracil for MCF-7 cells. Compounds **12** and **13** inhibited thymidylate synthase enzyme, with an half maximal inhibitory concentration, IC_50_ of 2.52 µM and 4.38 µM, while a standard drug, pemetrexed, showed IC_50_ = 6.75 µM. The molecular docking data of compounds **12** and **13** were found to be in support of biological activities data. In conclusion, hybrids (**12** and **13**) may inhibit thymidylate synthase enzyme, which could play a significant role as a chemotherapeutic agent.

## 1. Introduction

Cancer is uncontrolled cell growth and cell proliferation, and remains a global burden despite the advancements in cancer diagnosis and treatment [1]. The available anticancer drugs on the market develop resistance against the chemotherapeutic agents and toxicity to normal cells [2,3]. Chemotherapy is the only effective treatment, causing the inhibition of cancer cell growth and induction of apoptosis, as the DNA levels in tumor cells are significantly higher than in normal cells [4,5]. Interestingly, thymidylate synthase (TS) has now emerged as an important target for chemotherapy for cancer treatment, as it is directly involved in DNA synthesis [6].

Thymidylate synthase enzyme catalyzes the reductive methylation of deoxyuridine monophosphate (dUMP) to deoxythymidine monophosphate (dTMP) and 5,10-methylenetetrahydrofolate (CH_2_THF) [7,8]. This dTMP upon phosphorylation changes into thymine triphospate (dTTP), which acts as a direct precursor for the synthesis of DNA [9,10]. The blocking of dTMP causes a decrease in deoxythymidine triphosphate (dTTP), thereby interrupting DNA biosynthesis causing DNA damage [11]. In addition, TS inhibition causes an increase in dUMP, leading to surge in the deoxyuridine triphosphate (dUTP) level [12]. One of the anticancer drugs, 5-fluoruracil (5-FU), acts as a strong thymidylate inhibitor for various cancers [13]. Also, a 5-FU metabolite, fluorodeoxyuridine monophosphate (FdUMP), binds with a TS active site to form a stable ternary complex, thus blocking the binding of the dUMP with TS and leading to the inhibition of dTMP synthesis [14]. 

**Figure 1:**
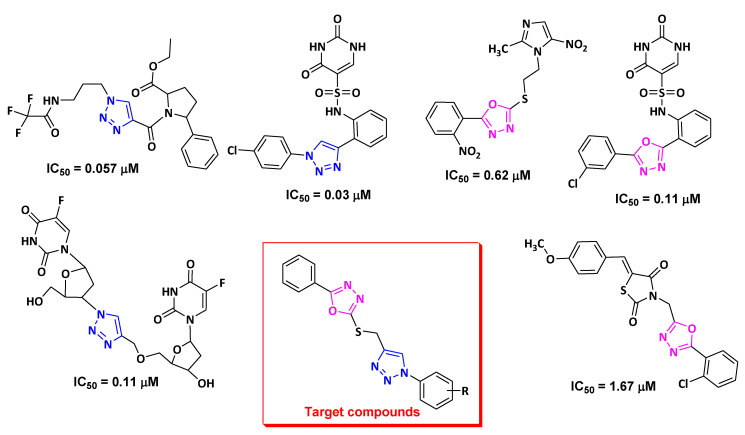
Reported thymidylate synthase inhibitors containing 1,3,4-oxadiazole and 1,2,3-triazoles.

Heterocycle has been a main pharmacophore in drug discovery and development. In the last few years, there is an emergence in the development of different 1,2,3-triazole-linked heterocycles, due to their excellent pharmacological properties, including anticancer [15], antiviral [16], antidiabetic [17], antimicrobial [18], anti-inflammatory [19], and antitubercular [20]. Compounds containing 1,2,3-triazoles have been reported to exert anticancer effect by inhibiting TS enzymes [21,22,23]. On the other hand, 1,3,4-oxadiazole plays a crucial part in medicinal chemistry. Zibotentan, an anticancer drug containing 1,3,4-oxadiazole as a pharmacophore, is used for various cancers [24]. Furthermore, 1,3,4-oxadiazole-linked heterocycles have also been reported to act as potential TS inhibitors [25,26]. Therefore, combining these two heterocycles in one molecule may lead to development of effective TS inhibitor (Figure 1).

In our previous work, we have reported thiazolidinedione-linked 1,3,4-oxadiazole as a promising TS inhibitor [27]. In continuation of our work to develop an effective TS inhibitor, we tried to conjugate 1,3,4-oxadiazole and 1,2,3-triazole pharmacophore in a single hybrid, which can inhibit the TS enzyme effectively. In this work, we describe the synthesis, pharmacokinetic study, anticancer, and TS inhibitory activities. The active compounds were docked against the thymidylate synthase enzymes to see the molecular interactions of active compounds with binding proteins. 

## 2. Results and Discussion

### 2.1. Chemistry

**Figure 2:**
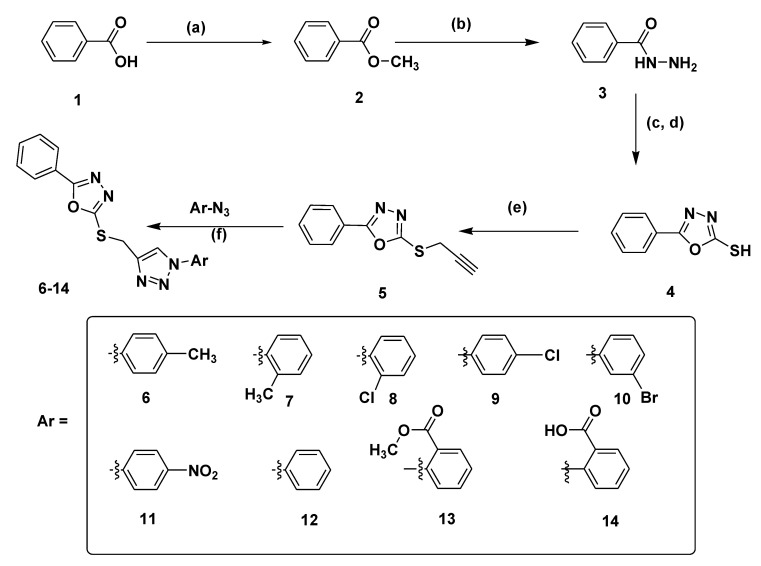
Synthesis of 1,3,4-oxadiazole linked 1,2,3-triazole hybrids. Reagents and conditions: (**a**) Methanol, Conc. H_2_SO_4_, reflux, 6h; (**b**) Absolute ethanol, H_2_N-NH_2_.H_2_O, reflux 4h; (**c**) Absolute ethanol, CS_2_, KOH, stir, 24 h; (**d**) reflux, 14 h, Conc. HCl; (**e**) Acetone, K_2_CO_3_, Propargyl bromide, stir, 50–60 °C, 6h; (**f**) tert.butanol:water (1:1), CuSO_4_.5H_2_O, sodium ascorbate, stir, 6–12 h.

Esterification of benzoic acid **1** in the presence of MeOH and concentrated H_2_SO_4_ yielded methyl benzoate **2**, which further reacted with hydrazine monohydrate in absolute ethanol to give acid hydrazide **3**. To this acid hydrazide **3** were added ethanol, carbon disulphide, and KOH, and the mixture was stirred for 24 h and then refluxed for 14 h. After completion of the reaction, monitored by Thin Layer Chromatography (TLC), the reaction mixture was concentrated, and 100 mL cold water was added. The acidification of aqueous solution with concentrated HCl (pH 3–4) produced a white precipitate, which was filtered and recrystalized in ethanol to afford compound **4.** The propargylation of compound **4** in the presence of potassium carbonate and propargyl bromide yielded the key intermediate **5.** Finally, the key intermediate **5**, using a click chemistry approach with different aromatic azides in the presence of copper sulphate pentahydrate and sodium ascorbate, yielded final compounds **6**–**14** (Figure 2).

All the synthesized conjugates have been confirmed using different analytical techniques, such as IR, NMR, and mass spectrometry. Formation of the compounds **2** and **3** were done by the presence of the molecular ion peak at 137 in Electron Spray Ionisation (ESI ) mass spectra and from their boiling point and melting point, respectively [28]. Formation of compound **4** was done by the presence of stretching frequency of an oxadiazole ring at 1500–1600 cm^−1^,–SH bond stretching at 2400 cm^−1^ in the IR spectrum, presence of an S–H singlet at δ 10.77 ppm in the ^1^H NMR spectrum, and the molecular ion signal at δ 179 [M + H]^+^ in mass spectrum. The propargylation of compound **4** to compound **5** was confirmed by the absence of chemical shift of an –SH proton at δ 10.77 ppm, as well as the presence of a triplet at δ 2.34 ppm and a singlet at δ 4.5 ppm for terminal alkyne proton and S–CH_2_- protons, respectively. Finally, the formation of the compound **5** was supported by the presence of a molecular ion signal at 217 [M + H] ^+^ in the ESI mass spectrum. Structural confirmation of final hybrids **6**–**14** was observed by disappearance of terminal alkyne proton peak at δ 2.34 ppm, as well as the appearance of a 1,2,3-triazole ring proton in the range of δ 8.19–8.89 ppm with additional aromatic protons. Finally, the presence of molecular ion peaks in the mass spectrum confirmed the formation of all the final compounds (The spectra are provided in Supplementary Materials).

### 2.2. Pharmacokinetics Studies/ADME Predictions

Nowadays, in silico pharmacokinetic predictions are extensively used in drug discovery to reduce the cost and time. To be orally available, the molecule is not only required to have excellent biological activity, but it must follow the desired pharmacokinetic properties. The in silico studies that have been carried out on synthesized molecules **6**–**14** have been to check whether these molecules satisfy the desirable pharmacokinetics or not, which plays a crucial role in drug discovery. The physicochemical properties directly affect the pharmacokinetic behavior. The synthesized molecules should obey the Lipinski [29] and Veber [30] rules for an orally available drug. The molecule must follow the following paremeters: molecular weight (MW) should be less than 500, the number of hydrogen bond acceptors (HBAs) should be fewer than 10, the number of hydrogen bond donors (HBD) should be fewer than 5, the partition coefficient (logP) should be less than 5, and the number of rotatable bonds should be ≤10 for drug likeness and good bioavailability (Table 1). 

From the above data, it was noticed that the synthesized molecules **6**–**14** follow the desired pharmacokinetic properties. All the final compounds, except compound **11**, showed high gastrointestinal absorption. The % absorption of the molecules was found to be in the range 60.44–76.24. Compounds **6, 7, 8, 9, 10**, and **12** showed the highest absorption of 76.24%, while compound **11** showed the lowest absorption at 60.44%. All the compounds followed Lipinki and Veber rules, i.e., molecular weight ranging from 335–414, hydrogen bond acceptors ranging from 5–7, hydrogen bond donors ranging from 0–1, lipophilicity appearing in the range 2.48–3.62, and the number of rotatable bonds between 5 and 7. The synthesized compounds exhibited higher logP values, in the range 2.48–3.62, suggesting higher cell membrane permeability. The final hybrids exhibited topological polar surface areas (TPSAs) between 94.93–140.75 A², which suggests good intestinal absorption. The pharmacokinetic results indicate that these compounds satisfy the criteria for good drug likeness parameters and good bioavailability.

### 2.3. Biological Studies

#### 2.3.1. Cytotoxicity Assay

The final compounds **6**–**14** were screened for their antiproliferative effect using an MTT (3-(4,5-dimethylthiazol-2-yl)-2,5-diphenyl tetrazolium bromide) assay. These compounds were tested against breast MCF-7 and colorectal HCT-116 cell lines. These compounds exhibited moderate to significant activity compared to standard drugs tamoxifen and 5-fluorouracil (5-FU). The results are presented in Table 2. 

**Figure 3:**
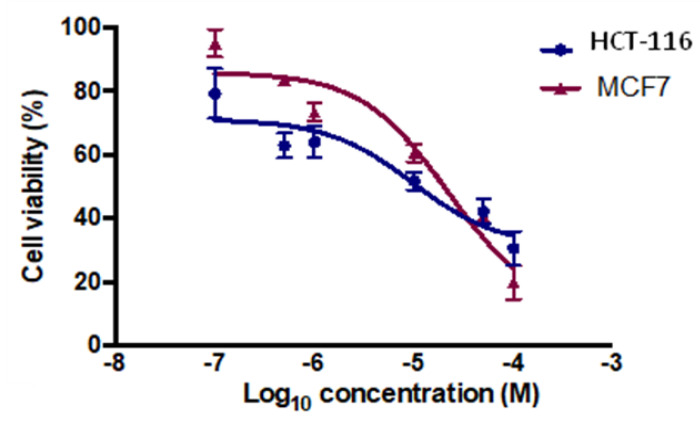
Antiproliferative effects of compound **12** in HCT-116 and MCF-7 cells. Cell viability was expressed as a percentage of vehicle control (ethanol 1% (*v*/*v*)) and was measured by MTT (3-(4,5-dimethylthiazol-2-yl)-2,5-diphenyl tetrazolium bromide) assay. The values represent the mean ± standard error of the mean (SEM) for three independent experiments performed in triplicate.

**Figure 4:**
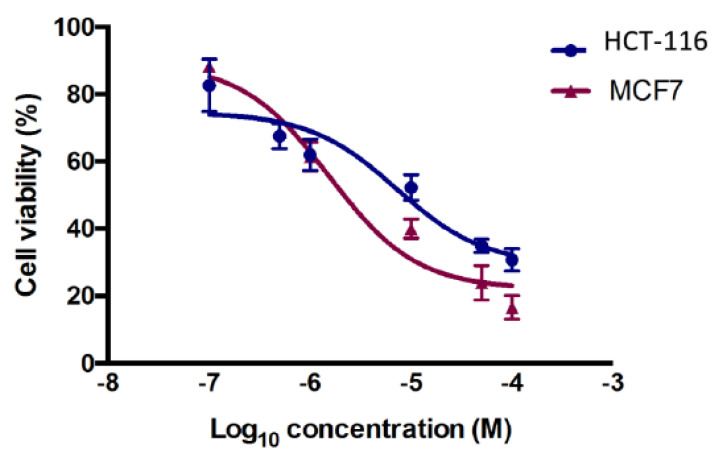
Antiproliferative effects of compound **13** in HCT-116 and MCF-7 cells. Cell viability was expressed as a percentage of vehicle control (ethanol 1% (*v*/*v*)) and was measured by MTT assay. The values represent the mean ± SEM for three independent experiments performed in triplicate.

Among the tested compounds, compounds **12** and **13** showed significant inhibitory effects on the viability of MCF-7 and HCT-116 cells (Figure 3). Compound **12** (IC_50_ = 5.8 µM) showed comparable inhibition to the standard drug tamoxifen (IC_50_ = 5.12) µM and four-fold inhibition compared to the standard drug 5-fluoruracil (IC_50_ = 24.74 µM). Compound **13**, however, was found to exhibit promising inhibition against MCF-7, with IC_50_ = 1.26 µM, while the two standard drugs, tamoxifen and 5-florouracil, showed IC_50_ = 5.12 µM and 24.74 µM, respectively. It is clear from the results that compound **13** was more potent in exerting the anticancer effect, with five times the activity of tamoxifen and 24 times the activity of 5-fluorouracil for MCF-7 (Figure 4). 

The same two compounds (**12** and **13**) also displayed significant inhibition against HCT-116 cells. Compound **12** (IC_50_ = 14.8 µM) revealed 1.7-fold activity compared to tamoxifen (IC_50_ = 25.41 µM) and 2.2-fold activity compared to 5-fluorouracil (IC_50_ = 32.68 µM) against HCT-116, while compound **13** (IC_50_ = 17.3 µM) displayed 1.9-fold activity compared to 5-FU and 1.5-fold the activity of tamoxifen. Other compounds, **6, 7, 8, 9, 10, 11**, and **14**, exhibited moderate cytotoxicity with the MCF-7, with IC_50_ values in the range 25.9–98.28 µM. On the other hand, compounds **6, 7, 9, 10,** and **14** displayed inhibition with IC_50_ values in the range of 32.7–89.2 µM, whereas compounds **8** and **11** were found to be inactive (IC_50_ > 100) against the HCT-116 cells. 

**Figure 5:**
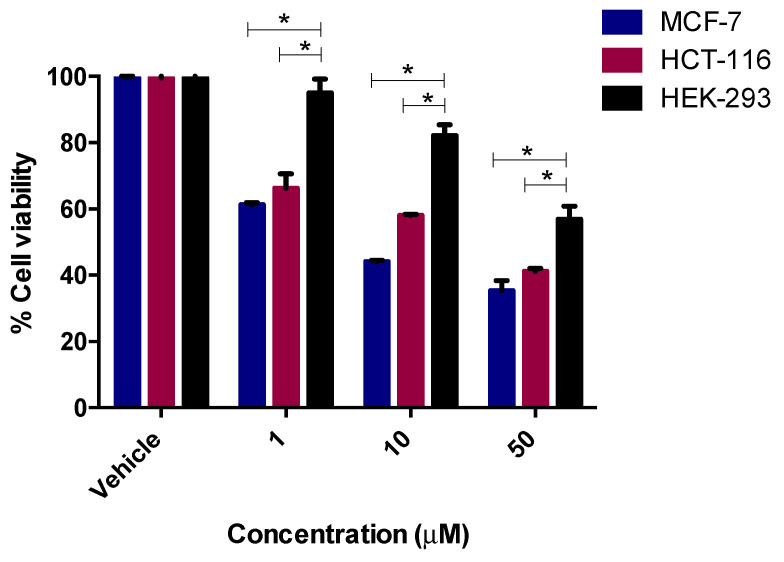
Antiproliferative effects of compound **12** on the viability of cancerous MCF-7 and HCT-116 cells, as well as non-tumorigenic HEK-293 cells. The values represent the mean ± SEM for three independent experiments performed in triplicate. * *p* < 0.05 between the indicated groups, via two-way ANOVA (Bonferroni post-test).

**Figure 6:**
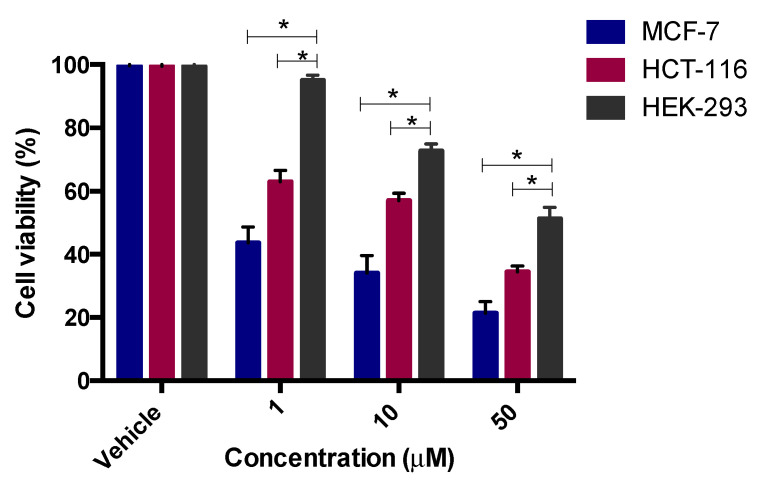
Antiproliferative effects of compound **13** on the viability of cancerous MCF-7 and HCT-116 cells, as well as non-tumorigenic HEK-293 cells. The values represent the mean ± SEM for three independent experiments performed in triplicate. * *p* < 0.05 between indicate groups, via two-way ANOVA (Bonferroni post-test).

The most potent compounds, **12** and **13**, were also tested on non-tumorigenic cell line HEK 293 (normal human embryonic kidney) cells, in order to investigate the toxicity and selectivity of these two potent compounds (Figure 5 and Figure 6). It was found that these two compounds (**12** and **13**) showed IC_50_ values above 50 μM on HEK 293 cells, which was higher than those observed against the two cancer cell lines—MCF-7: IC_50_ = 5.8 μM (**12**), 1.26 μM (**13**); HCT-116: IC_50_ = 14.8 μM (**12**), 17.3 μM (**13**)—indicating that these molecules was less toxic to human normal cells and selective to cancerous cells. From these results, it is clear that compound **12** with no substituents and compound **13** with a –COOMe group at the ortho position exerted promising antiproliferative effects on both the tested cells, whereas the presence of halogens and electron withdrawing groups (nitro) on the aromatic ring did not played a significant role in exerting the anticancer effect.

#### 2.3.2. In Vitro Thymidylate Synthase Activity

**Figure 7:**
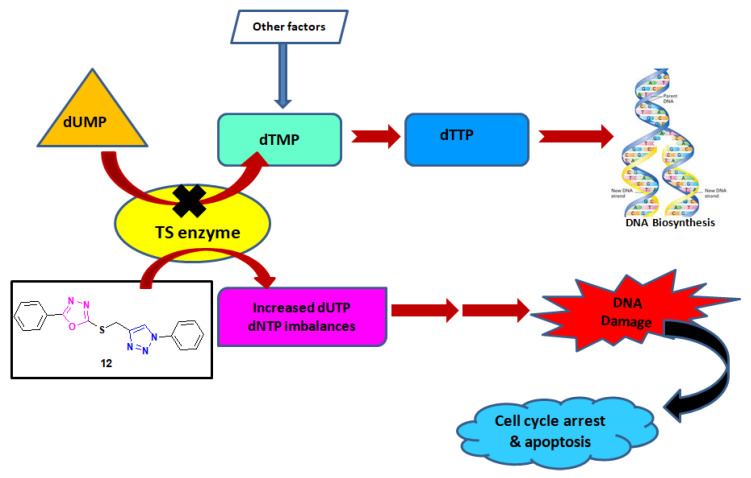
Mechanism of action of compound **12** on thymidylate synthase enzyme.

Thymidylate synthase (TS) has become an important target for cancer treatment, as it is involved in DNA synthesis. The inhibition of this enzyme plays a vital role in chemotherapy treatment. The active compounds (**12** and **13**) from the MTT assay were screened for in vitro thymidylate synthase, to confirm its mechanism of action. These compounds inhibited the TS enzyme significantly compared to the standard drug pemetrexed. Compound **12** inhibited TS with IC_50_ = 2.52 µM, and **13** inhibited it with IC_50_ = 4.38 (Table 3). It was observed that compounds **12** and **13** showed 2.6- and 1.5-fold activity compared to pemetrexed (IC_50_ = 6.75 µM). From these results, it can be inferred that compounds **12** and **13** inhibit TS by binding with the active binding site of the enzyme, which results in the blocking of dUMP with TS, leading to inactivation of dTMP. This inactivation of dTMP results in a reduction of deoxythymidine triphosphate (dTTP), leading to the disruption of DNA synthesis and cessation of cell growth and proliferation (Figure 7). These results support our antiproliferative results.

### 2.4. Molecular Docking

**Figure 8:**
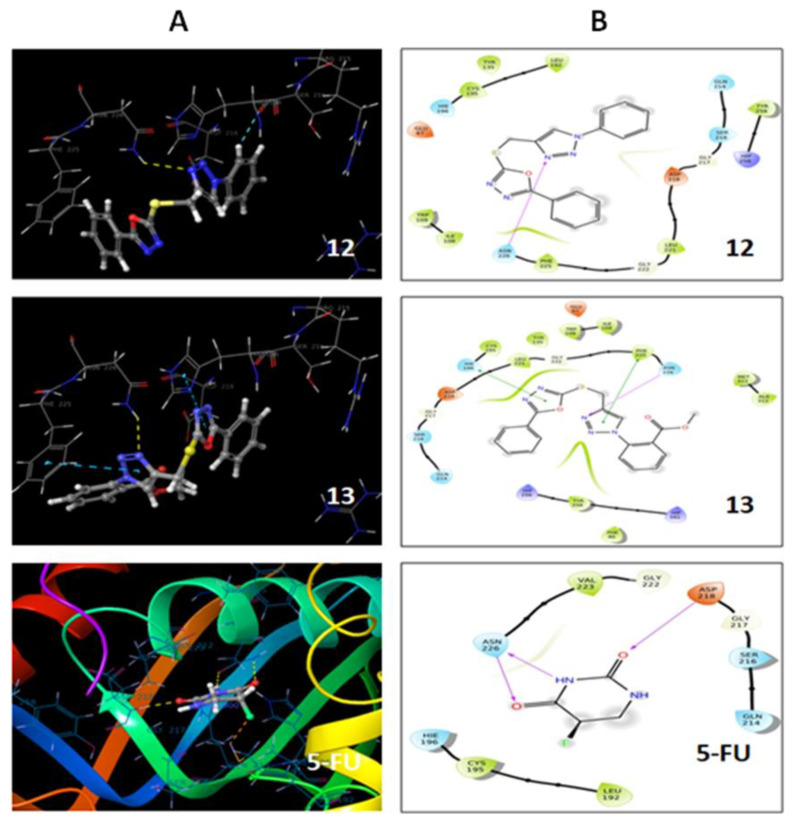
Molecular docking of the active compounds **12** and **13**, as well as 5-FU, against thimidylate synthase (TS) protein 6QXG. (**A**) Binding mode of **12**, **13**, and 5-FU at the TS binding site, with a three-dimensional (3D) plot. (**B**) Binding mode of **12**, **13**, and 5-FU at the TS binding site on a two-dimensional (2D) plot. 5-FU: 5-fluorouracil.

It is a computational technique used frequently to know the possible interactions of a molecule with a receptor. The active compounds from in vitro studies were docked against TS proteins (PDB = 6QXG) to support our in vitro thymidylate synthase results, as well as to know the possible binding modes with the protein. The 5-fluorouracil has been reported as a TS inhibitor that interacts with the active binding site of the protein through different residues [31,32]. Therefore, we have docked our active compounds **12** and **13** against this protein, in order to support our in vitro findings.The results are presented in Figure 8 and Table 4.

From the docking results, it was observed that compound **12** (−3.81) and **13** (−4.25) showed higher dock scores than the standard drug 5-fluorouracil (−3.5). The nitrogen of the 1,2,3-triazole ring in compounds **12** and **13** showed hydrogen bonding interactions with ASN 226 residues, while two pi–pi interactions were also observed in compound **13**. One pi interaction was between a 1,2,3-oxadiazole ring and HIE 196, and another was the interaction of a 1,2,3-triazole ring with a PHE 225 residue. The standard drug 5-FU showed H-bonding interactions of ASP 218 with a C=O group at the 2 position, and ASN 226 with N–H and a C=O group at the 3 and 4 positions. The binding pattern of compounds **12** and **13**, as well as 5-FU, was found to be similar to the thymidylate synthase protein. These finding suggests that these two compounds (**12** and **13**) exert anticancer effects by inhibiting the thymidylate synthase enzyme, which supports the in vitro TS results of hybrids **12** and **13**, with IC_50_ values of 2.52 and 4.38 µM, respectively.

## 3. Experimental

### 3.1. Chemistry

The chemicals and other reagents were procured from Sigma Aldrich (Germany) and Loba Chem (India), and were used without further purification. FTIR spectra were recorded on a Thermo Scientific iS 50 by ATR method. Nuclear magnetic resonance (NMR) analysis was performed on Bruker 300 and 850 MHz instruments in CDCl_3_ or DMSO-d_6_ solvents; tetramethylsilane (TMS) was used as an internal reference. Chemical shift and coupling constant are provided in parts per million (ppm) and Hertz, respectively. All the samples were analyzed by mass spectrometry on a Thermo Scientific-LCQ Fleet (LCF10605) using the electron spray ionization method, and are provided in *m*/*z*. The melting points of the compounds were determined on the electro-thermal melting point apparatus (Stuart SMP40). A LEECO Elementar Analyzer was used for elemental analysis of the synthesized compounds, which was reported in % standard; these were ±0.4% of the calculated values. Monitoring of the reaction and purity of the compounds were checked on a silica gel G plate (Merck, Germany). 

### 3.2. General Procedure for the Synthesis of 1,3,4-Oxadiazole Linked 1,2,3-Triazole Hybrids (6–14)

Compound **5** (0.01 mmole) was charged in a 100 mL round-bottom flask, followed by addition of tert-BuOH–H_2_O (1:1, 30–50 mL). The reaction mixture was heated to get a clear solution and cooled to room temperature. To this reaction mixture, copper sulphate pentahydrate (0.0013 mmol) and sodium ascorbate (0.0013 mmol) was added and stirred for 30 mins, followed by drop-wise addition of aromatic azides (0.15 mmole). The reaction mixture was stirred for 5–12 hrs at room temperature, and the progress of the reaction was monitored by TLC, using *n*-hexane/ethylacetate (6:4) as eluents. After completion of the reaction, water (50 mL) was added to the reaction mixture and the products were extracted with dichloromethane (100 mL × 2). The dichloromethane layer was combined, dried over anhydrous sodium sulphate, concentrated, and recrystallized using dichloromethane and cyclohexane to get target compounds **6**–**14** in 65–85% yield. 

#### 3.2.1. 4-((5-phenyl–1,3,4-oxadiazol–2-ylthio)methyl)-1-p-tolyl–1H-1,2,3-triazole (6) 

Grey crystals, yield = 80%, melting point (MP) = 146–147 °C. IR (ATR) ν_max_: 3086 (C–H), 1559 (C=C), 1518 1468, 1340, 1257, 1232 (C=N, C–N), 1192, 1079 (C–O), 700, 688 (C–S) cm^−1^. ^1^H NMR (300 MHz, CDCl_3_): δ 2.40 (s, 3H), 4.69 (s, 2H), 7.28 (d, *J* = 8.1 Hz, 2H), 7.46–7.59 (m, 5H), 7.98 (d, *J* = 7.2 Hz, 2H), 8.19 (s, 1H). ^13^C NMR (75 MHz, CDCl_3_): δ 21.01, 27.25, 120.41, 122.48, 123.49, 126.87, 129.86, 130.68, 132.49, 134.68, 138.86, 143.91, 163.40, 165.84. ESI MS: 350 (M^+^ + H). C_18_H_15_N_5_OS (calculated): C = 61.87; H = 4.33; N = 20.04; O = 4.58; S = 9.18; observed: C = 61.42; H = 4.35; N = 20.01; O = 4.60; S = 9.16.

#### 3.2.2. 4-((5-phenyl–1,3,4-oxadiazol-2-ylthio)methyl)–1-o-tolyl-1H–1,2,3-triazole (7)

White powder, yield = 75%, MP = 112–113 °C. IR (ATR) ν_max_: 3123 (C–H aromatic), (C–H), 2920 (C–H), 1606, 1557 (C=C), 1505, 1491, 1466, 1382, 1341 (C=N, C–N), 1185, 1076 (C–O), 701, 683 (C–S) cm^−1^. ^1^H NMR (300 MHz, CDCl_3_): δ 2.52 (s, 3H), 4.75 (s, 2H), 7.11–7.14 (m, 2H), 7.57–7.66 (m, 3H), 7.77–8.0 (3, 4H), 8.72 (s, 1H). ^13^C NMR (75 MHz, CDCl_3_): δ 17.75, 27.33, 123.51, 125.94, 126.41, 126.89, 127.43, 129.86, 130.31, 131.80, 132.50, 133.47, 136.57, 142.86, 163.36, 165.87. ESI MS: 350 (M^+^ + H). C_18_H_15_N_5_OS (calculated): C = 61.87; H = 4.33; N = 20.04; O = 4.58; S = 9.18; observed: C = 61.82; H = 4.36; N = 20.01; O = 4.60; S = 9.17.

#### 3.2.3. 4-((5-phenyl–1,3,4-oxadiazol–2-ylthio)methyl)–1-(2-chlorophenyl)-1H–1,2,3-triazole (8)

White crystals, yield = 65%, MP = 111–112 °C. (ATR) ν_max_: 3142, 3089 (C–H aromatic), 1557 (C=C), 1490, 1470, 1342 (C=N, C–N), 1192, 1073, 1064 (C–O), 769, 717 (C–Cl), 704, 686 (C–S) cm^−1^. ^1^H NMR (300 MHz, CDCl_3_): δ 4.71 (s, 1H), 7.39–7.61 (m, 7H), 7.97–8.00 (m, 2H), 8.23 (s, 1H). ^13^C NMR (75 MHz, CDCl_3_): δ 27.19, 123.49, 126.51, 126.89, 128.77, 128.85, 128.92, 129.86, 130.99, 132.13, 132.50, 134.80, 142.98, 163.31, 165.87. ESI MS: 370 (M^+^ + H), 372 (M^+^ + 2 + H). C_17_H_12_N_5_OS (calculated): C = 55.21; H = 3.27; N = 18.94; O = 4.33; S = 8.67; observed: C = 55.16; H = 3.30; N = 18.89; O = 4.35; S = 8.64.

#### 3.2.4. 4-((5-phenyl–1,3,4-oxadiazol-2-ylthio)methyl)–1-(4-chlorophenyl)-1H–1,2,3-triazole (9)

Brown powder, yield = 75%, MP = 200–202 °C. (ATR) ν_max_: 3082 (C–H, aromatic), 2920 (C–H), 1558 (C=C), 1490, 1473, 1448, 1427, 1339 (C=N, C–N), 1195, 1093, 1077, 1051 (C–O), 846, 827 (C–Cl), 702, 686 (C–S) cm^−1^. ^1^H NMR (300 MHz, CDCl_3_): δ 4.68 (s, 2H), 7.26–7.28 (m, 5H), 7.49 (t, *J* = 7.8 Hz, 2H), 7.67 (d, *J* = 8.7 Hz, 2H), 7.87 (d, *J* = 6.6 Hz, 2H), 8.24 (s, 1H). ^13^C NMR (75 MHz, CDCl_3_): δ 27.18, 119.06, 120.30, 122.77, 123.47, 126.83, 128.97, 129.81, 132.00, 132.45, 134.64, 137.92, 144.23, 163.33, 165.82. ESI MS: 370 (M^+^+H), 372 (M^+^+2+H). C_17_H_12_N_5_OS (calculated): C = 55.21; H = 3.27; N = 18.94; O = 4.33; S = 8.67; observed: C = 55.16; H = 3.30; N = 18.93; O = 4.37; S = 8.63.

#### 3.2.5. 4-((5-phenyl–1,3,4-oxadiazol–2-ylthio)methyl)-1-(3-bromophenyl)-1H–1,2,3-triazole (10) 

White flakes, yield = 70%, MP = 146–148 °C. IR (ATR) ν_max_: 3137, 3083 (C–H, aromatic), 1607, 1588 (C=C), 1558, 1486, 1471, 1461, 1341, 1289, 1255 (C=N, C–N), 1191, 1077, 1046 (C–O), 702 (C–S), 691 (C–Br) cm^−1^. ^1^H NMR (300 MHz, CDCl_3_): δ 4.67 (s, 2H), 7.35–7.68 (m, 6H), 7.92–8.00 (m, 3H), 8.24 (s, 1H). ^13^C NMR (75 MHz, CDCl_3_): δ 27.19, 119.53, 122.79, 122.89, 123.09, 123.50, 126.87, 129.86, 131.95, 132.28, 132.49, 138.02, 144.22, 163.32, 165.85. ESI MS: 414 (M^+^ + H), 416 (M^+^ + 2 + H). C_17_H_12_N_5_OS: (calculated): C = 49.29; H = 2.92; N = 16.90; O = 3.86; S = 7.74; observed: C = 49.31; H = 2.94; N = 16.87; O = 3.83; S = 7.76.

#### 3.2.6. 4-((5-phenyl–1,3,4-oxadiazol–2-ylthio)methyl)–1-(4-nitrophenyl)-1H–1,2,3-triazole (11) 

Light orange solid, yield = 65%, MP = 202–204 °C. IR (ATR) ν_max_: 3127, 3079 (C–H, aromatic), 1596 (C=C), 1558 (N–O) 1523, 1505, 1473, 1388 (C=N, C–N), 1260, 1232, 1191, 1109 (C–O), 703, 686 (C–S) cm^−1^. ^1^H NMR (850 MHz, DMSO–d_6_) δ: 4.79 (s, 2H), 7.27–8.01 (m, 9H), 8.26 (s, 1H). ^13^C NMR (213 MHz, DMSO) δ: 27.25, 120.17, 12.87, 123.38, 126.60, 127.47, 127.66, 128.54, 128.70, 131.98, 132.22, 137.11, 147.54, 163.52, 165.58. ESI MS: 381 (M + H). C_17_H_12_N_6_O_3_S (calculated): C = 53.68; H = 3.18; N = 22.09; O = 12.62; S = 8.43; observed: C = 53.69; H = 3.20; N = 22.07; O = 12.62; S = 8.42.

#### 3.2.7. 4-((5-phenyl–1,3,4-oxadiazol–2-ylthio)methyl)-1-phenyl-1H–1,2,3-triazole (12) 

White flakes, yield = 75%, MP = 130–131 °C. IR (ATR) ν_max_: 3144 (C–H), 1594 (C=C), 1506, 1466, 1344, 1290, 1256 (C=N, C–N), 1191, 1173, 1077, 1064 (C–O), 702, 683 (C–S) cm^−1^. ^1^H NMR (300 MHz, CDCl_3_): δ 4.69 (s, 2H), 7.36–8.24 (m, 10H), 8.35 (s, 1H).^13^C NMR (75 MHz, CDCl_3_): δ 27.26, 120.55, 122.58, 123.51, 126.86, 129.21, 129.85, 130.34, 132.48, 136.92, 143.99, 163.37, 165.85; ESI MS: 336 (M^+^ + H). C_17_H_13_N_5_OS (calculated): C = 60.88; H = 3.91; N = 20.88; O = 4.77; S = 9.56; observed: C = 60.81; H = 3.95; N = 20.85; O = 4.80; S = 9.55.

#### 3.2.8. Methyl 2-(4-((5-phenyl–1,3,4-oxadiazol-2-ylthio)methyl)-1H–1,2,3-triazol–1-yl)benzoate (13) 

Brown flakes, yield = 70%, MP = 119–120 °C. IR (ATR) ν_max_: 3127, 3083 (C–H, aromatic), 2953 (C–H), 1727 (C=O), 1602, 1558 (C=C), 1506, 1472, 1450, 1340, 1272 (C=N, C–N), 1192, 1134, 1053 (C–O), 702, 688 (C–S) cm^−1^. ^1^H NMR (300 MHz, CDCl_3_): δ 3.85 (s, 3H), 4.68 (s, 2H), 6.99 (d, *J* = 9.0 Hz, 2H), 7.21–7.75 (m, 5H), 7.93–8.44 (m, 2H), 8.45 (s, 1H). ^13^C NMR (75 MHz, DMSO) δ:27.30, 56.00, 115.32, 122.21, 122.51, 122.83, 123.51, 126.86, 129.84, 130.34, 132.46, 143.66, 163.40, 165.84, 169.77. ESI MS: 394 (M^+^ + H). C_19_H_15_N_5_O_3_S (calculated): C = 58.01; H = 3.84; N = 17.80; O = 12.20; S = 8.15; observed: C = 58.02; H = 3.82; N = 17.81; O = 12.21; S = 8.14.

#### 3.2.9. 2-(4-((5-phenyl–1,3,4-oxadiazol–2-ylthio)methyl)-1H–1,2,3-triazol–1-yl)benzoic acid (14) 

White solid, yield = 72%, MP = 119–120 °C. IR (ATR) ν_max_: 3127 (brs–OH) 3083, 2953 (C–H), 1727 (C = O), 1602, 1558 (C=C), 1506, 1472, 1450, 1340, 1272 (C=N, C–N), 1260, 1192, 1134, 1053 (C–O), 702, 688 (C–S) cm^−1^.^1^H NMR (400 MHz, DMSO): δ 4.69 (s, 2H), 7.13–7.24 (m, 2H), 7.41–7.99 (m, 7H), 8.89 (s, 1H), 10.66 (s, 1H).^13^C NMR (213 MHz, DMSO): δ 27.26, 120.01, 125.48, 126.76, 132.57, 134.09, 135.61, 138.57, 143.77, 163.92, 165.21, 167.27; ESI MS: 380 (M^+^ + H). C_18_H_13_N_5_O_3_S (calculated): C = 56.98; H = 3.45; N = 18.46; O = 12.65; S = 8.45; observed: C = 56.99; H = 3.47; N = 18.43; O = 12.64; S = 8.45.

### 3.3. Anticancer Activity

#### 3.3.1. Cell Lines and Culture Medium

The human breast cancer cell line (MCF-7) used in the present study was obtained from Dr. Neamatallah’s lab. The cells were cultured in Dulbecco’s Modified Eagle Medium (DMEM) supplemented with 10% (*v*/*v*) fetal bovine serum (FBS), 10,000 units/mL penicillin/streptomycin, and 1% (*v*/*v*) *L*-glutamine at 37 °C in humidified 5% CO_2_ incubator. 

#### 3.3.2. Cytotoxicity Assay

The cytotoxicity activity was done by MTT assay [33]. Breast MCF-7 and colorectal HCT-116 cancer cells were added at 1 × 10^5^ cells/mL into a 96-well plate with three replicates, and incubated overnight for attachment at 37 °C in a 5% CO_2_ humidified atmosphere. Drug concentrations at six serial dilutions (100.0, 50.0, 10.0, 1.0, 0.5, and 0.1 μM) were added in triplicate and incubated at 37 °C and 5% CO_2_ for 72 h. Drugs were dissolved in 0.1% DMSO as a vehicle. Untreated cells were used as control. Tamoxifen and 5-fluorouracil (5-FU) was used as positive controls. Thereafter, each well for each time point was removed and replaced with 100 μM of full medium containing 10% 3-(4,5- dimethylthiaxolyl-2)-2,5-diphenyltetrazoliumbromide (MTT) (10 mg/mL). Then the media was removed and 100 µl of DMSO was added, and cells were incubated for a further 5 mins at 37 °C and 5% CO_2_. Plates were quantified using the SpectraMax M3 plate reader at 570 nm. The percentage inhibition was calculated as 100 − ((mean OD of treated cell × 100)/Mean OD of vehicle treated cells (DMSO)). All the experiments were repeated in at least three independent experiments (Table 2, Figure 3, Figure 4, Figure 5 and Figure 6).

### 3.4. In Vitro Thymidylate Synthase Enzyme Assay

A thymidylate synthase enzymatic assay was carried out according to the reported method [34,35]. It involves a mixture containing 2-mercaptoethanol (0.1 M), (6R,S)-tetrahydrofolate (0.0003 M), formaldehyde (0.012 M), MgCl_2_ (0.02 M), dUMP (0.001 M), TrisHCl (0.04 M), and NaEDTA (0.00075 M).This assay was done spectrophotometrically at 30° C and pH 7.4. The reaction was initiated by the addition of an amount of enzyme, giving a change in absorbance at 340 nm of 0.016/min in the absence of inhibitor. The percent inhibition was determined at a minimum of four inhibitor concentrations within 20% of the 50% point. The standard deviations for determination of the 50% points were within ± 10% of the values given. The results are presented in Table 3 and Figure 7. 

### 3.5. Molecular Docking

Molecular docking studies involve mainly protein selection and preparation, grid generation, ligand preparation, docking, and further analysis of docking studies. A protein with accession number 6QXG was selected and downloaded from Protein Data Bank. This protein is reported to act as a thymidylate synthase inhibitor. The protein was imported, optimized, and minimized by removing unwanted molecules and other defects reported by the software. The minimized protein was used for grid generation, which involves the selected ligand as the reference, as it signifies the binding sites of the drug with respect to the target. Molecules drawn in 3D form were refined by the LigPrep module. The molecules were subjected to an OPLS-2005 force field to generate a single, low-energy 3D structure for each input structure. Docking studies was carried using Glide software. It was carried out using extra precision and write XP descriptor information. This generates favorable ligand poses, which are further screened through filters to examine the spatial fit of the ligand in the active site. Ligand poses, which pass through an initial screening, are subjected to evaluation and minimization of grid approximation. Scoring was then carried on energy-minimized poses to generate a Glide score [36] (Table 4, Figure 8).

## 4. Conclusions

In the present article, a series of nine hybrids of 1,2,3-triazole and 1,3,4-oxadiazole moieties (**6**–**14**) have been described. The final compounds have been characterized using different analytical techniques. These hybrids have been tested for in vitro anticancer and thymidylate synthase activities. According to in silico pharmacokinetic studies, the synthesized hybrids exhibited good drug likeness properties and bioavailability. The cytotoxicity results indicated that compounds **12** and **13** exhibited remarkable inhibition on the tested MCF-7 and HCT-116 cell lines. Compound **12** showed four-fold inhibition compared to the standard drug 5-fluoruracil, and comparable inhibition to tamoxifen, whereas compound **13** exerted five-fold and 24 times the activity of tamoxifen and 5-fluorouracil, respectively, for MCF-7 cells. The same compounds (**12** and **13**) also revealed significant inhibition against HCT-116 cells. Compound **12** revealed 1.7-fold activity of tamoxifen and 2.2-fold activity of 5-fluorouracil, while compound **13** displayed 1.9-fold the activity of 5-FU and 1.5-fold that of tamoxifen against HCT-116. The in vitro thymidylate synthase activity results supported our cytotoxicity results. Compounds **12** and **13** inhibited thymidylate synthase enzyme with IC_50_ values of 2.52 µM and 4.38 µM, respectively, while the standard drug pemetrexed showed an IC_50_ of 6.75 µM. The molecular docking data of compounds **12** and **13** supported the in vitro biological activity data. In conclusion, hybrids (**12** and **13**) may inhibit the thymidylate synthase enzyme, which could play a significant role as a chemotherapeutic agent.

## Figures and Tables

**Table 1:** Pharmacokinetic/Absorption, Distribution, Metabolism and Elimination (ADME) predictions of the target compounds **6**–**14.**

No.	Lipinski Parameters					nROTB ^e^	TPSA ^f^	%ABS ^g^	BBB ^h^	GI ABS ^i^
	MW ^a^	HBAs ^b^	HBDs ^c^	LogP ^d^	Violations					
6	349.41	5	0	3.54	0	5	94.93	76.24	No	High
7	349.41	5	0	3.5	0	5	94.93	76.24	No	High
8	369.83	5	0	3.33	0	5	94.93	76.24	No	High
9	369.83	5	0	3.5	0	5	94.93	76.24	No	High
10	414.28	5	0	3.62	0	5	94.93	76.24	No	High
11	380.38	7	0	2.97	0	6	140.75	60.44	No	Low
12	335.38	5	0	3.21	0	5	94.93	76.24	No	High
13	393.42	7	0	3.39	0	7	121.23	67.17	No	High
14	379.39	7	1	2.48	0	6	132.23	63.38	No	High

^a^ Molecular weight; ^b^ hydrogen bond acceptors; ^c^ hydrogen bond donors; ^d^ partition coefficient; ^e^ number of rotatable bonds; ^f^ topological polar surface area; ^g^ absorption (%); ^h^ blood–brain barrier; ^i^ gastro-intestinal absorption.

**Table 2:** The IC_50_ (µM) of the synthesized compounds (**6**–**14**) against tested human cancer cell lines (MCF-7 and HCT-116).^a^

Compound	MCF-7 ^b^	HCT-116 ^c^
6	79.80	89.20
7	30.70	34.30
8	73.30	107.50
9	34.40	36.70
10	25.90	32.70
11	98.20	102.30
12	5.80	14.80
13	1.26	17.30
14	40.60	46.80
Tam ^d^	5.12	26.41
5-FU ^e^	24.74	32.68

^a^ IC_50_ values are the concentrations that cause 50% inhibition of cancer cell growth. Data represent the mean values ± standard deviation of three independent experiments, performed in triplicate; ^b^ breast cancer (MCF-7); ^c^ colorectal cancer (HCT-116); ^d^ Tam: tamoxifen; ^e^ 5-FU: 5-florouracil, which was used as a reference drug (positive control).

**Table 3:** In vitro thymidylate synthase (TS) activity of the active compounds **12** and **13**, as well as PTX.

Compounds	IC_50_ (µM)
12	2.52
13	4.38
PTX	6.75

IC_50_ values are the mean ± SD of three separate experiments. PTX: pemetrexed.

**Table 4:** Docking scores of active compounds **12** and **13** against human thymidylate synthase protein 6QXG.

Compound	Docking Score	Amino Acid Residue
12	−3.81	ASN 226
13	−4.25	ASN 226, PHE 225, HIE 196
5-FU	−3.5	ASP 218, ASN 226

## Supplementary Materials

The following are available online at https://www.mdpi.com/1424-8247/13/11/390/s1, Figures S1–S9: ^1^H NMR spectra of all final compounds; Figures S10–S18: ^13^C NMR spectra of all final compounds; Figures S19–S27: Mass spectra of all final compounds.
