# Supplementary material for: Synthesis of New 1, 3, 4-Oxadiazole-Incorporated 1, 2, 3-Triazole Moieties as Potential Anticancer Agents Targeting Thymidylate Synthase and Their Docking Studies
Source: Pharmaceuticals (Basel). 2020 Nov 14;13(11):390. doi: 10.3390/ph13110390 (PMC7696185; doi:10.3390/ph13110390)
Supplement: Supplementary file 1 [file pharmaceuticals-13-00390-s001.pdf]

SUPPLEMENTARY MATERIAL

Synthesis and molecular docking of new 1,3,4-oxadiazole incorporated 1,2,3-triazole moiety as a potential anticancer agent targeting thymidylate synthase.

Mohammad Mahboob Alam[1], Abdulraheem SA. Almalki [2], Thikryat Neamatallah [3,†], Nada M. Ali [1], Azizah M. Malebari [4] , Syed Nazreen [1,†,*]

[1] Department of Chemistry, Faculty of Science, Albaha University, Albaha, Kingdom of Saudi Arabia
[2] Department of Chemistry, Faculty of Science, Taif University, Taif, Kingdom of Saudi Arabia
[3] Department of Pharmacology and Toxicology, Faculty of Pharmacy, King Abdulaziz University, Jeddah, Kingdom of Saudi Arabia
[4] Department of Pharmaceutical Chemistry, Faculty of Pharmacy, King Abdulaziz University, Jeddah, Kingdom of Saudi Arabia

* Correspondence: syed.nazreen22885@gmail.com
† These authors contributed equally to this work

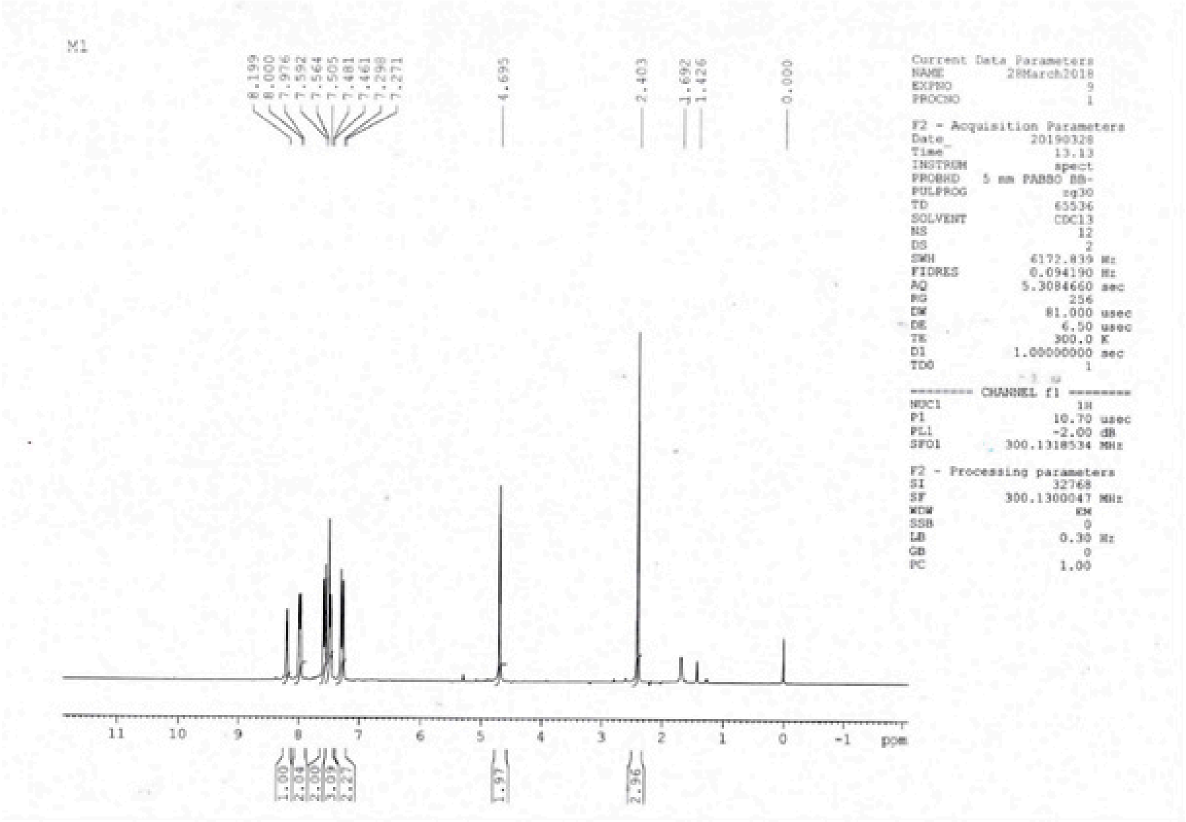

Figure S1: ^{1}H NMR of compound 6

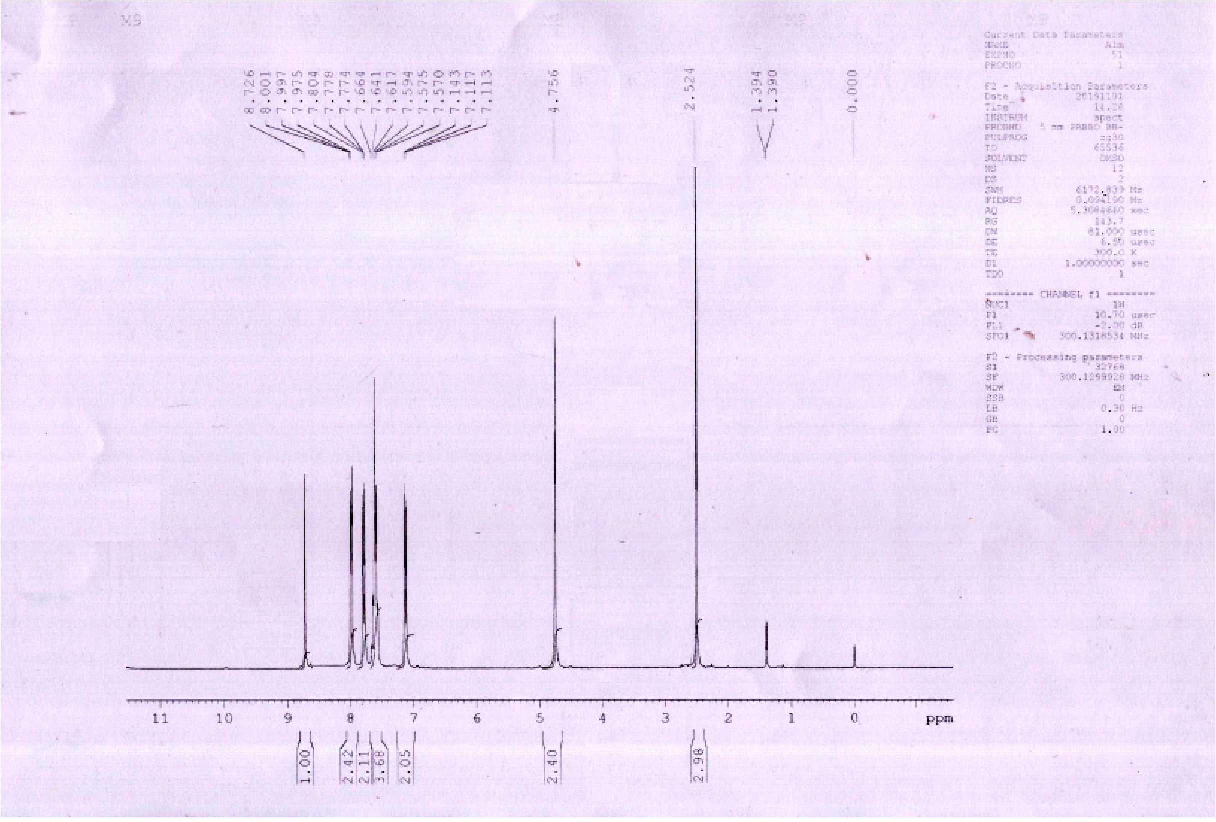

Figure S2: ^{1}H NMR of compound 7

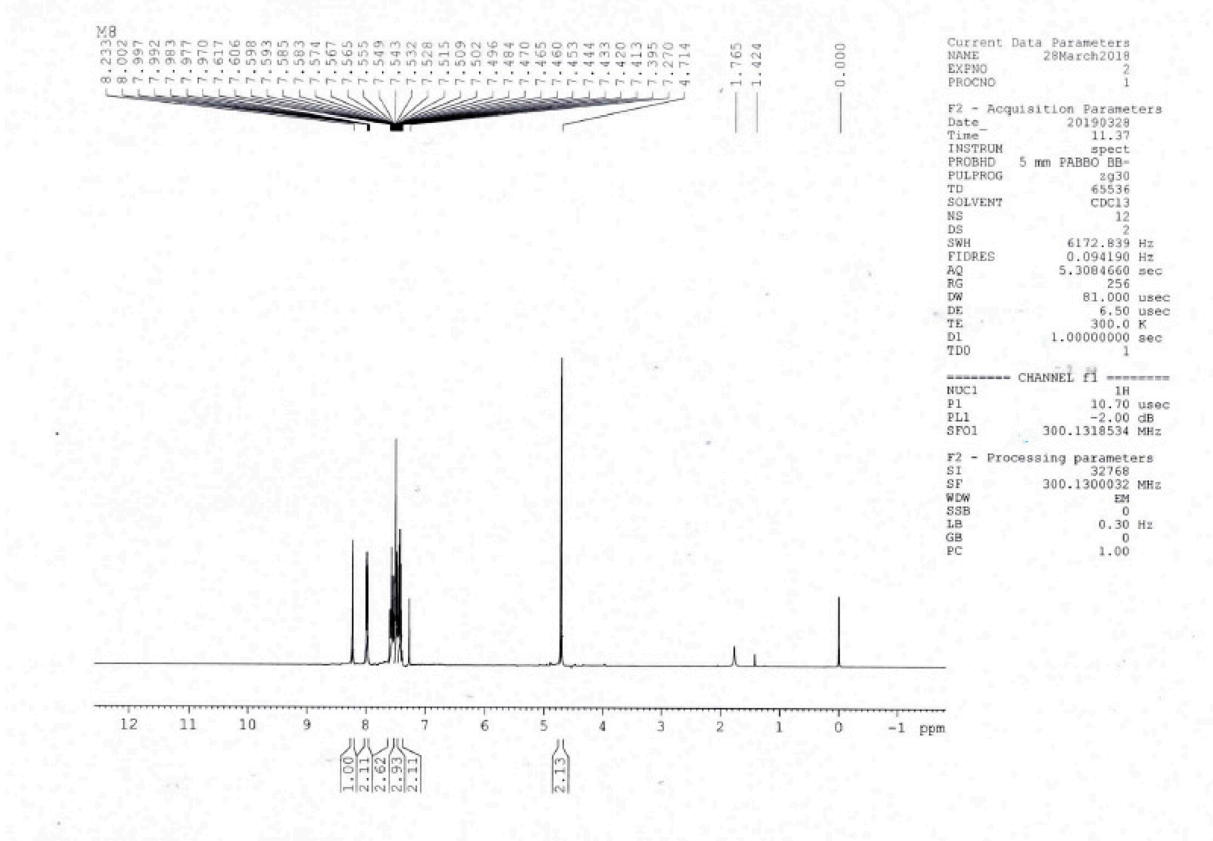

Figure S3: ^{1}H NMR of compound 8

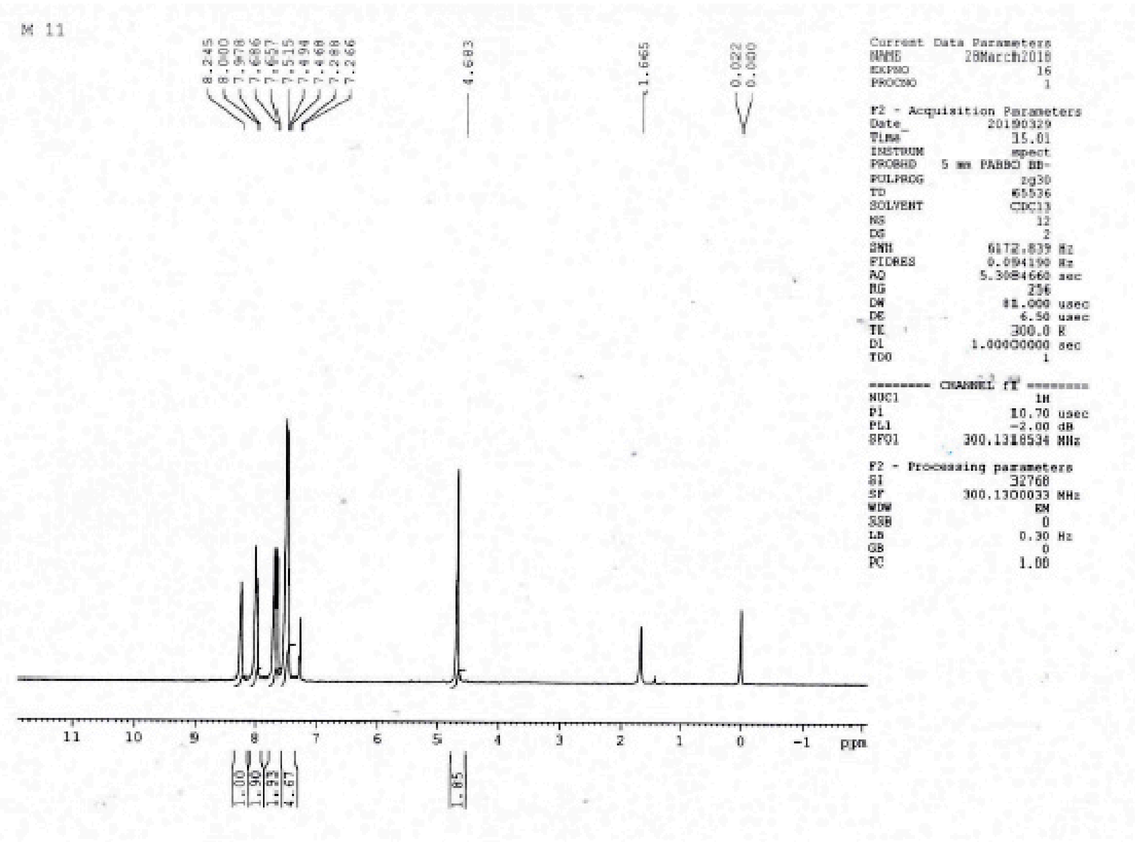

Figure S4: ^{1}H NMR of compound 9

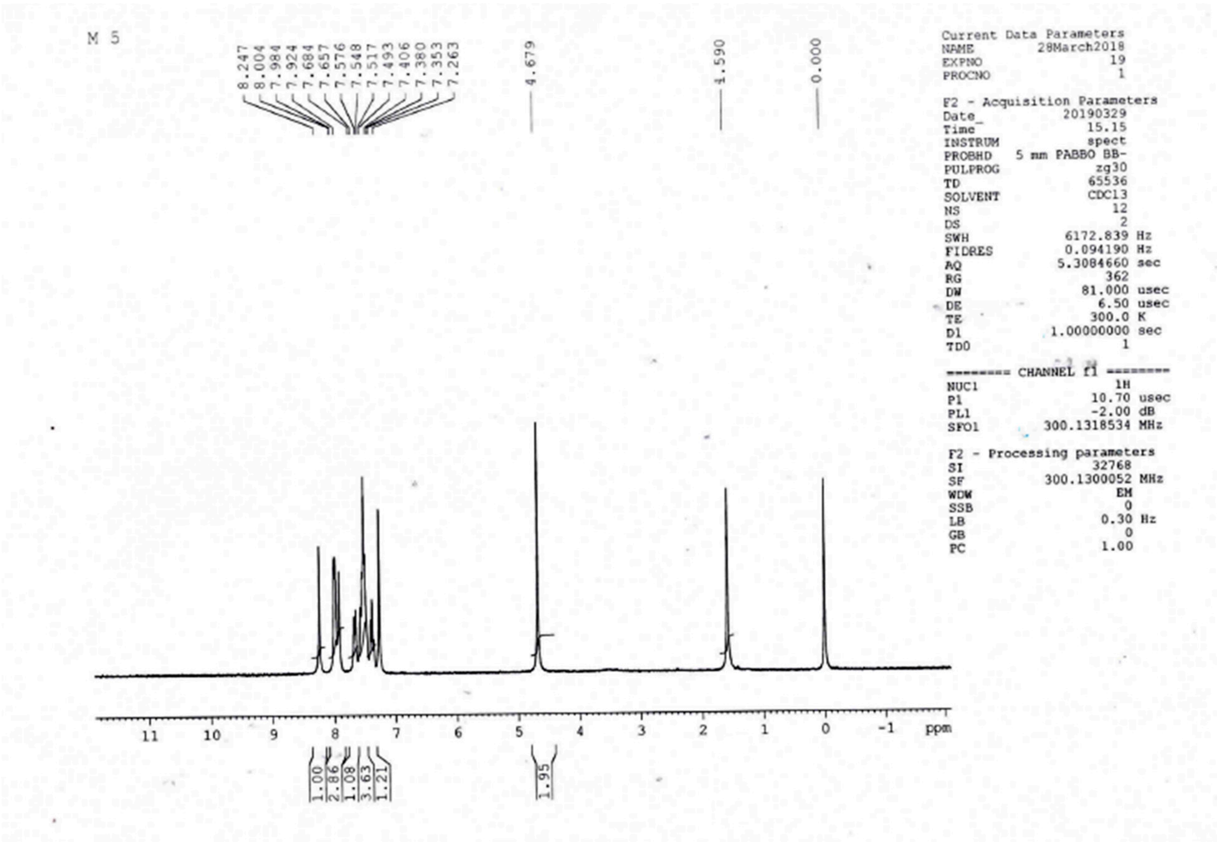

Figure S5: ^{1}H NMR of compound 10

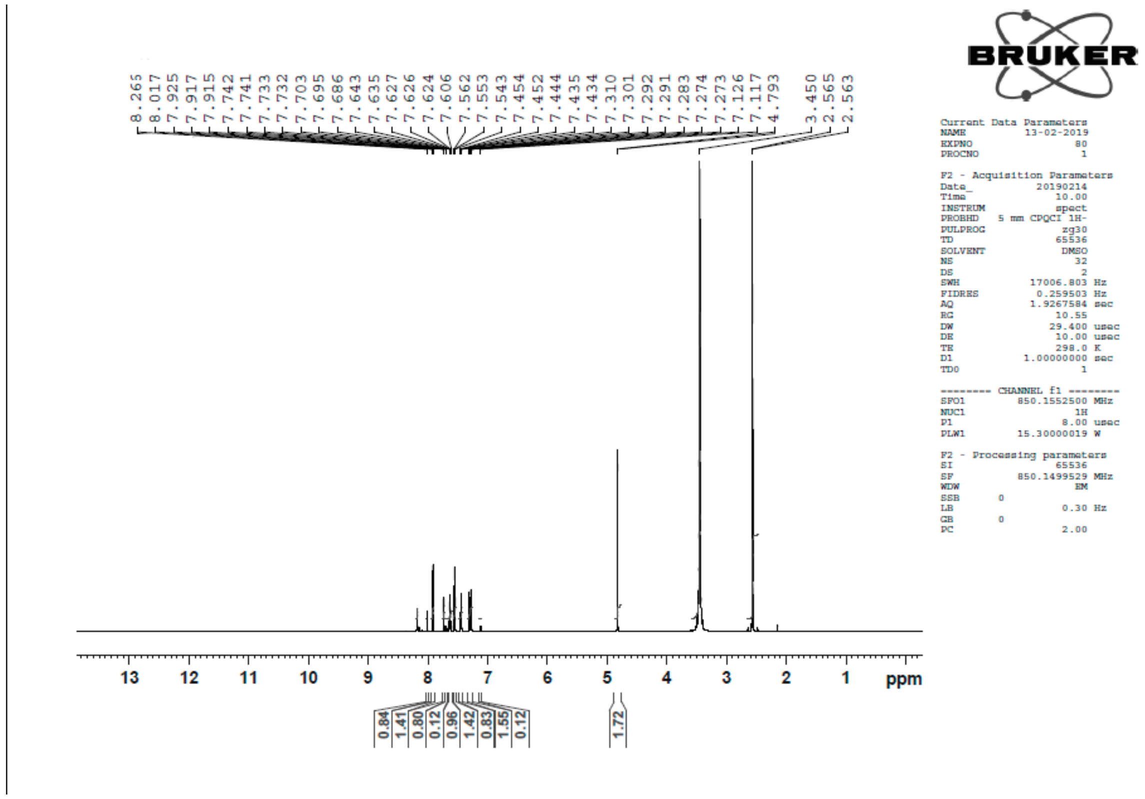

Figure S6: ^{1}H NMR of compound 11

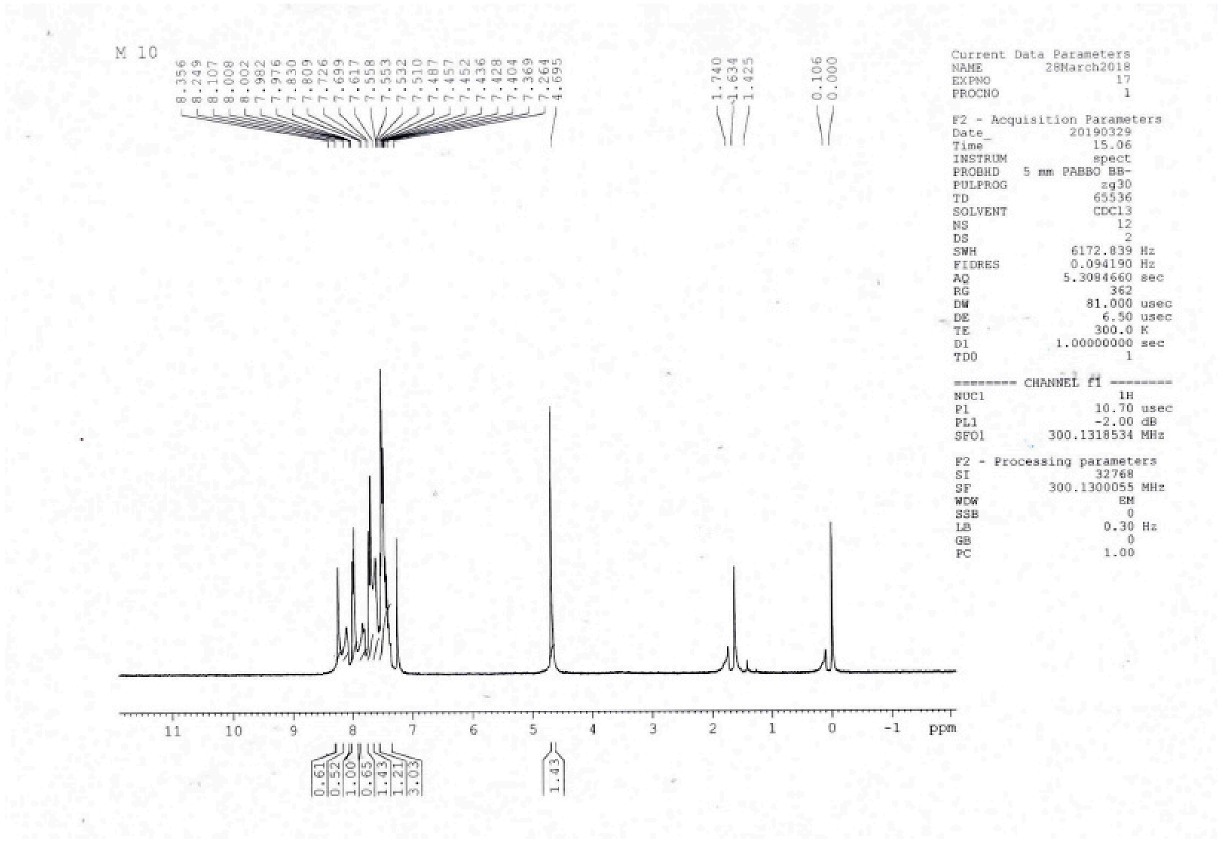

Figure S7: ^{1}H NMR of compound 12

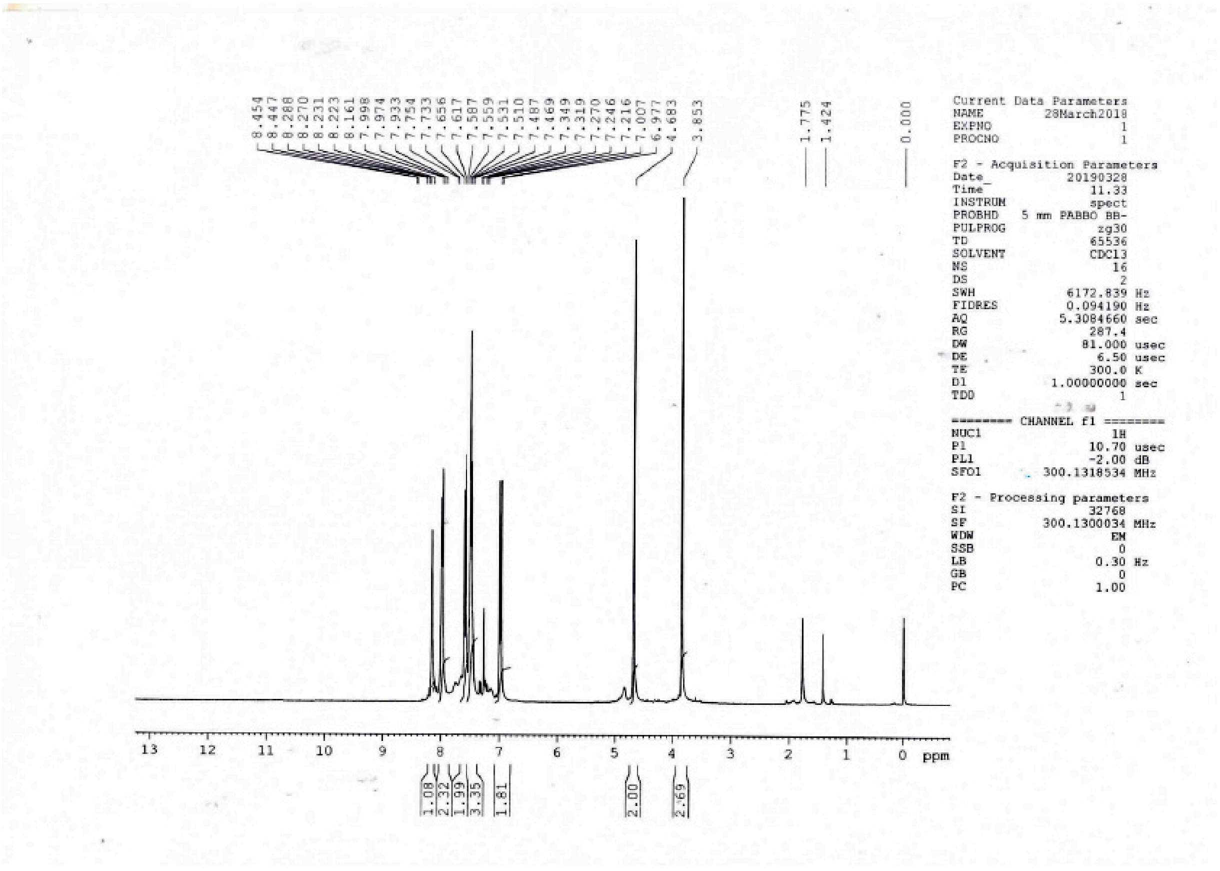

Figure S8: ^{1}H NMR of compound 13

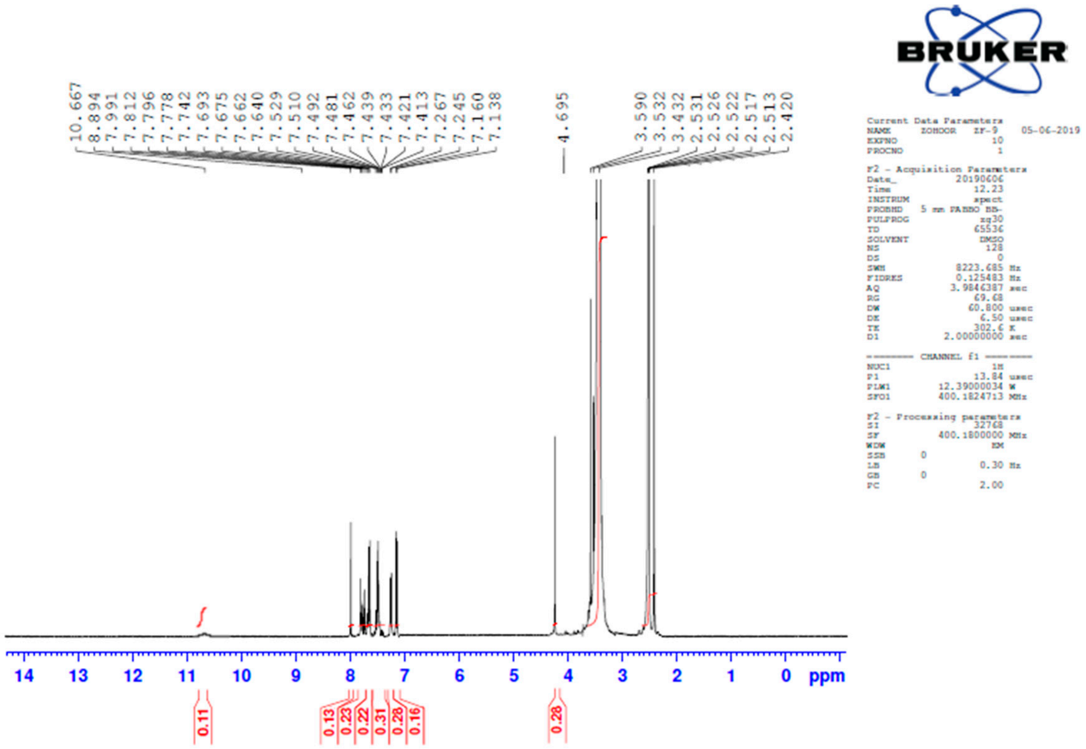

Figure S9: ^{1}H NMR of compound 14

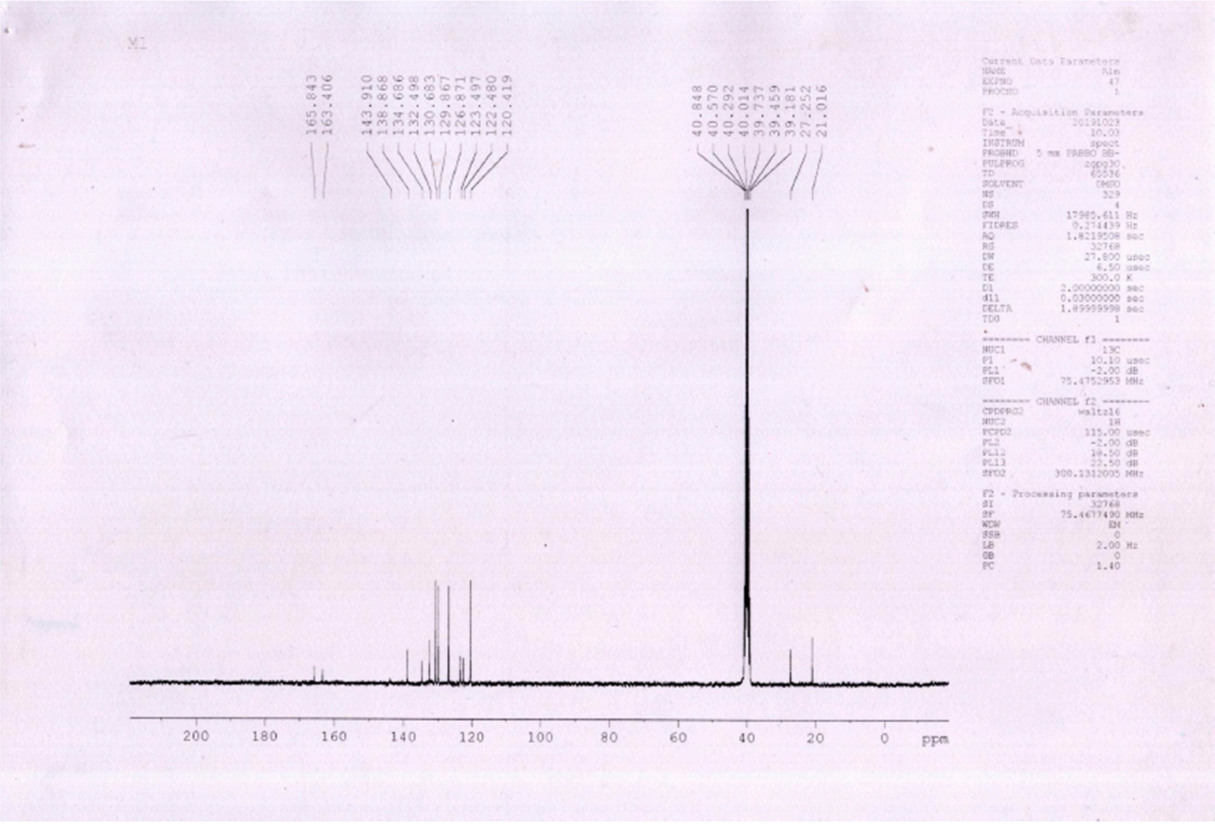

Figure S10:^{13}C NMR of compound 6

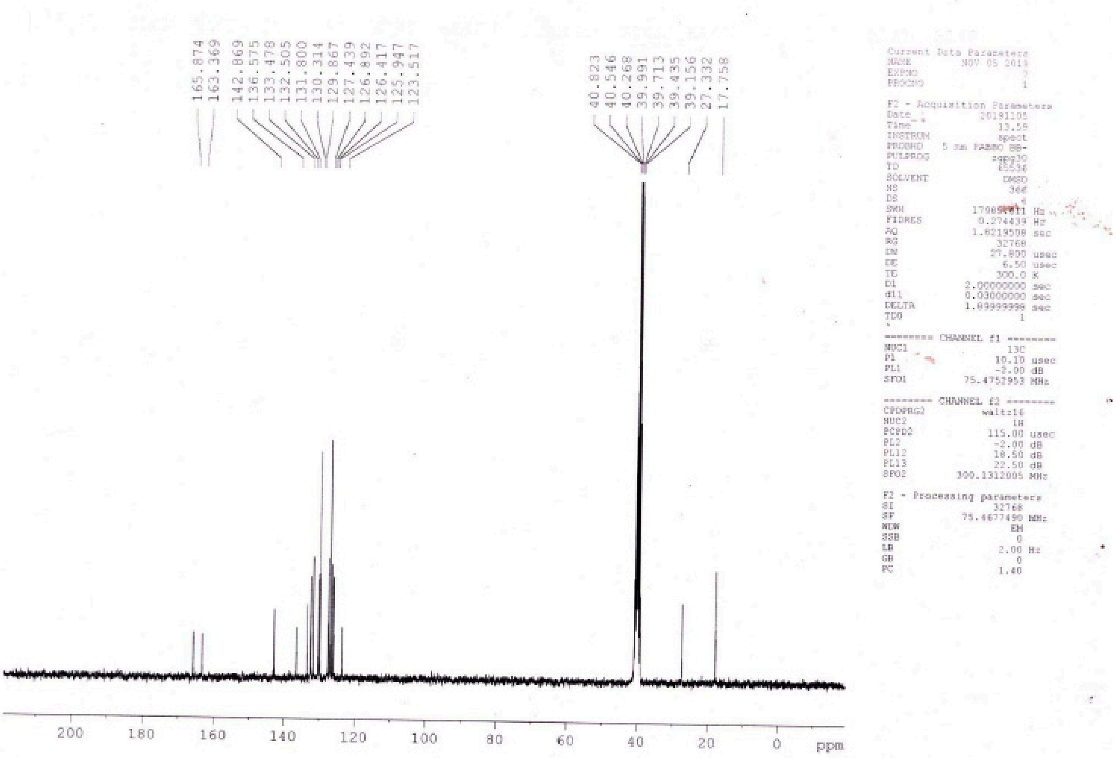

Figure S11:^{13}C NMR of compound 7

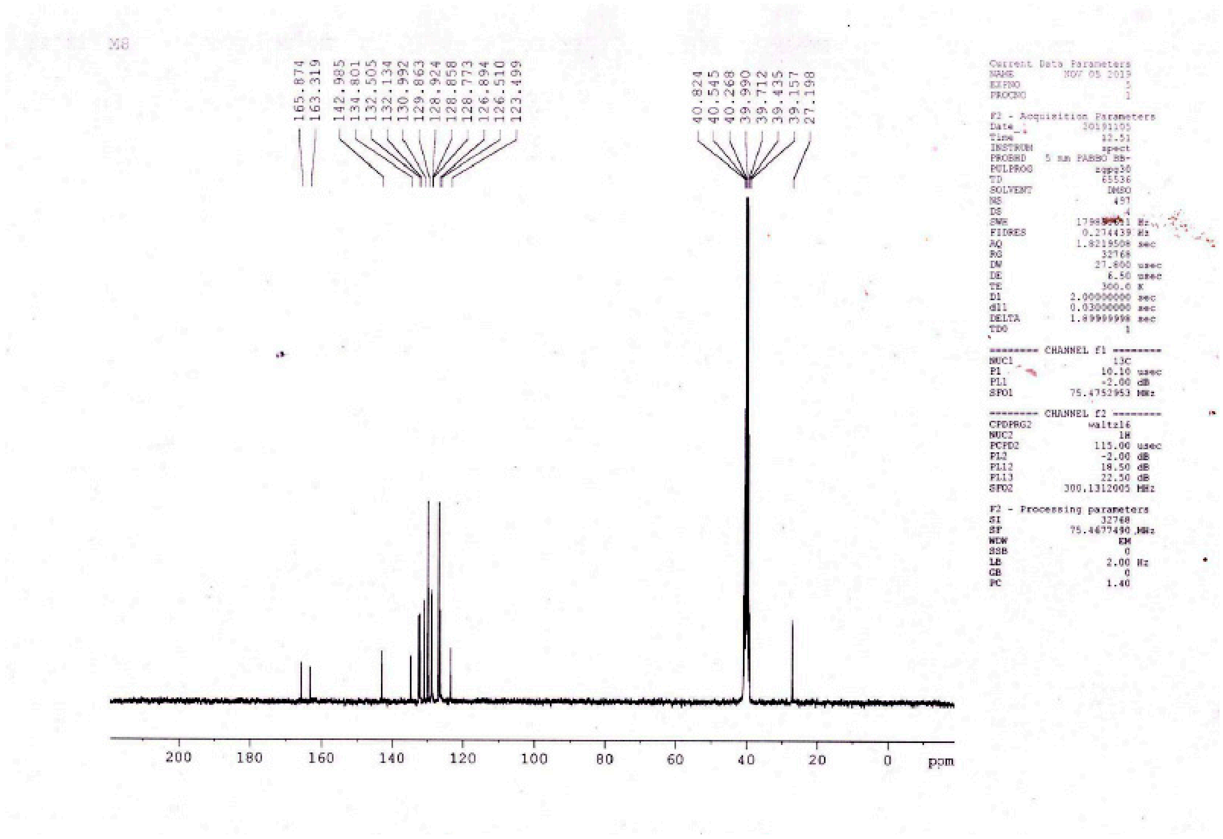

Figure S12:^{13}**C NMR of compound 8**

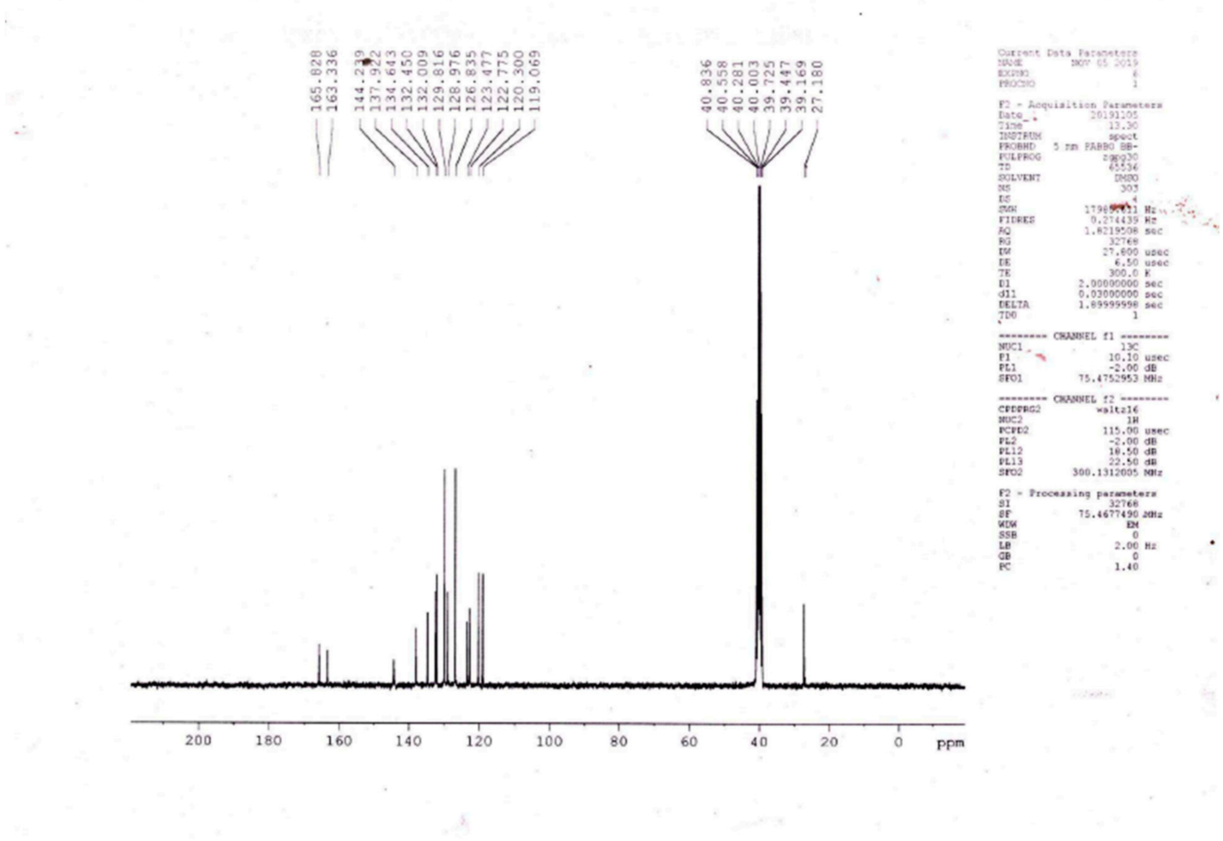

Figure S13:^{13}C NMR of compound 9

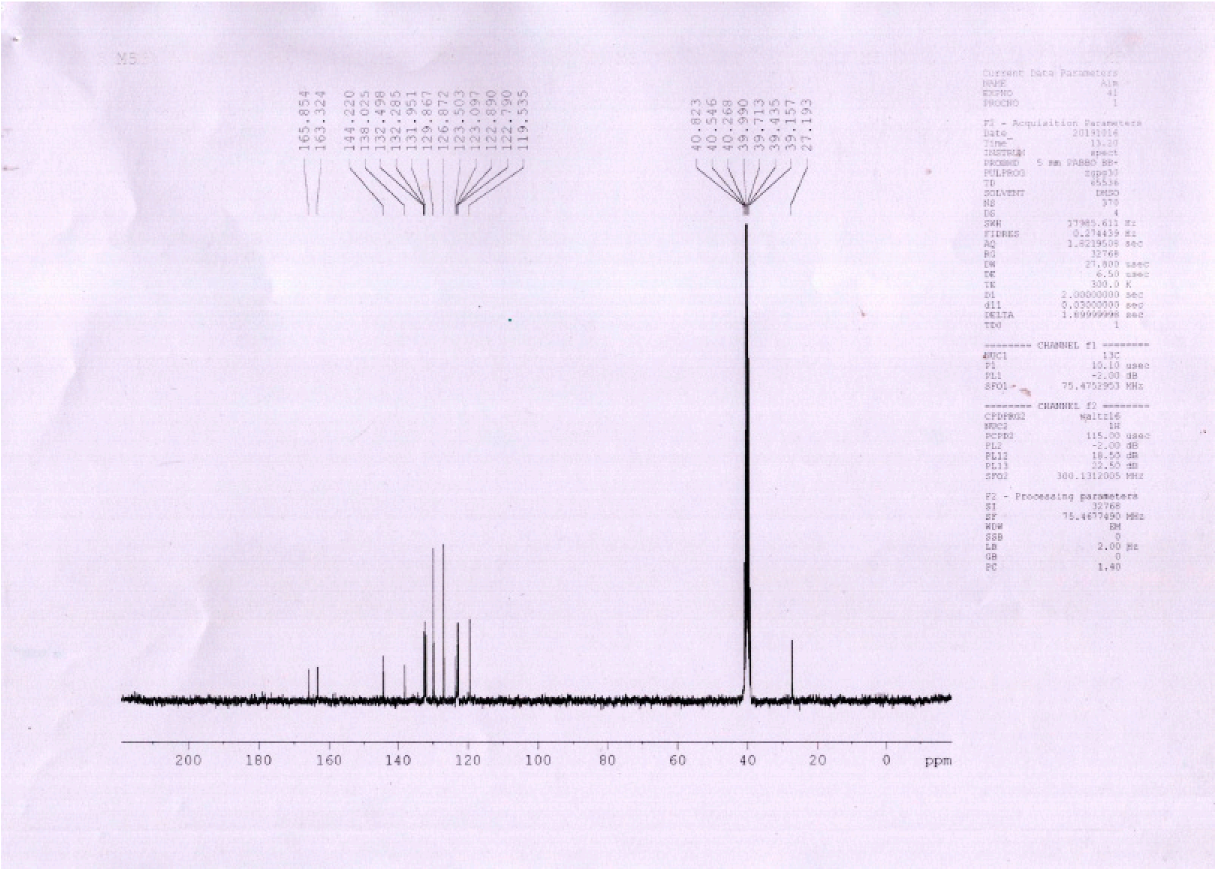

Figure S14:^{13}C NMR of compound 10

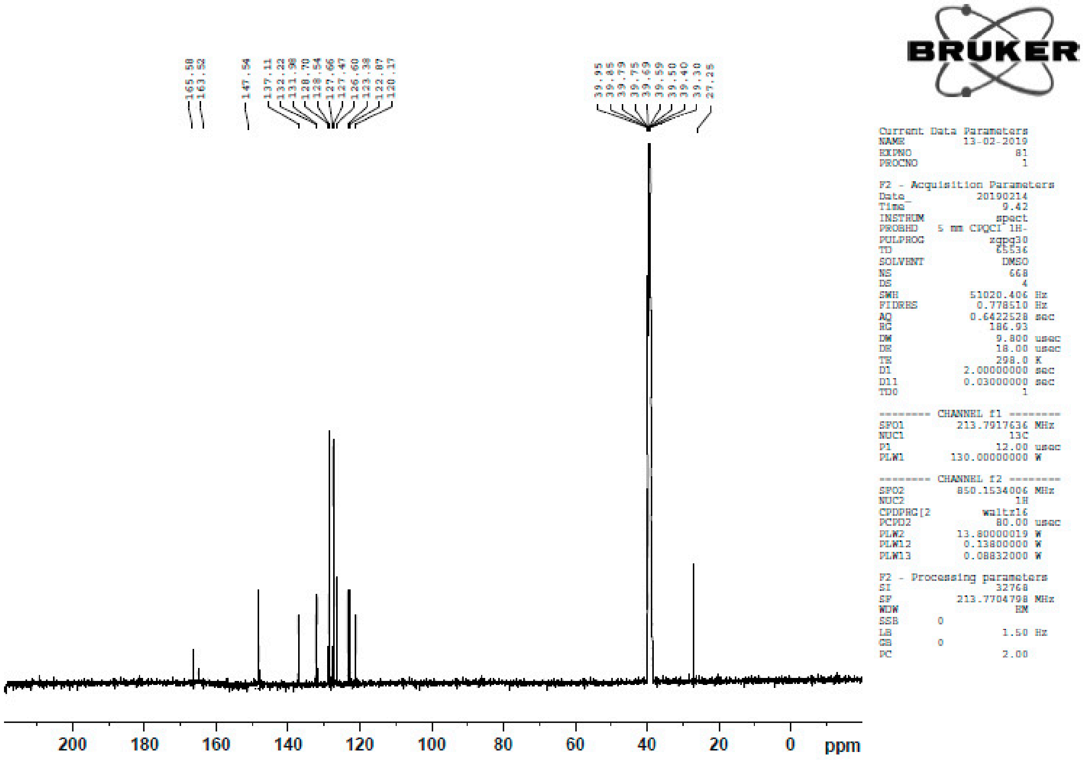

Figure S15:^{13}C NMR of compound 11

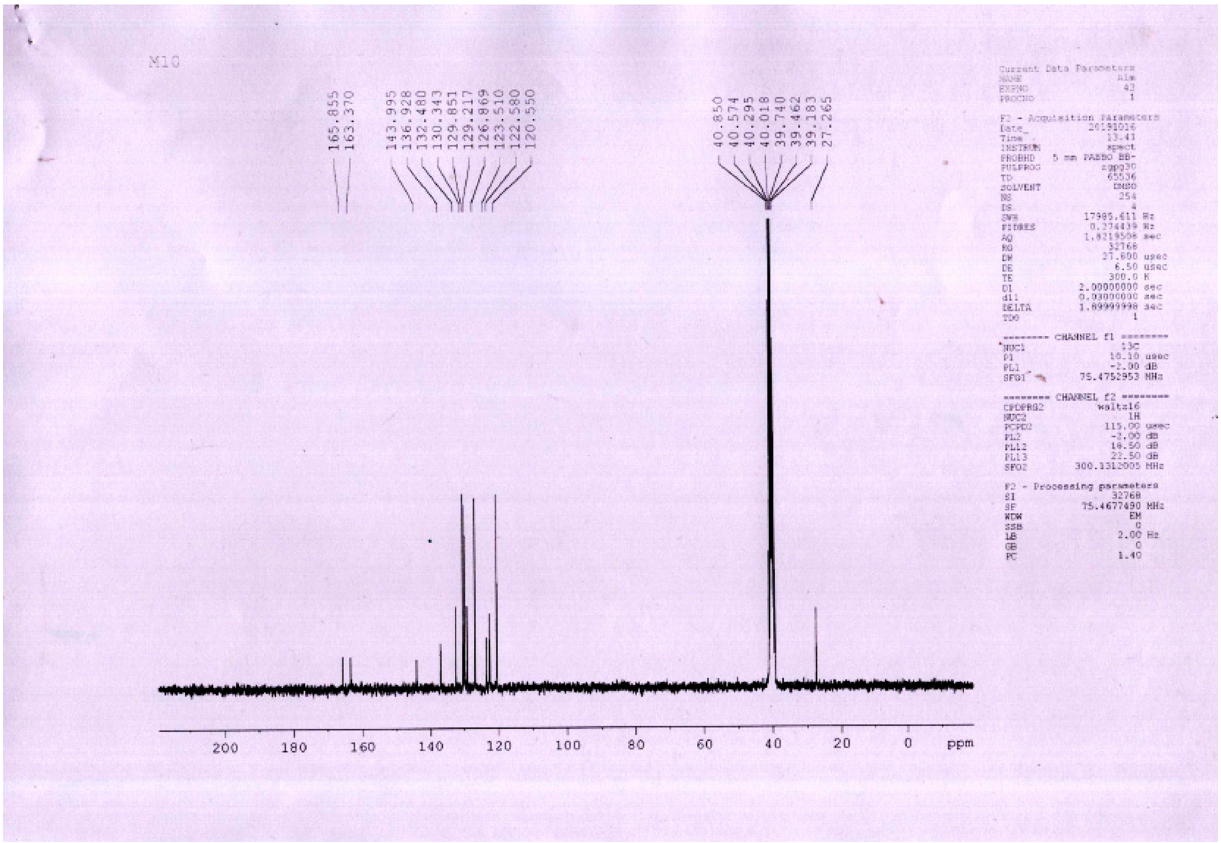

Figure S16:^{13}C NMR of compound 12

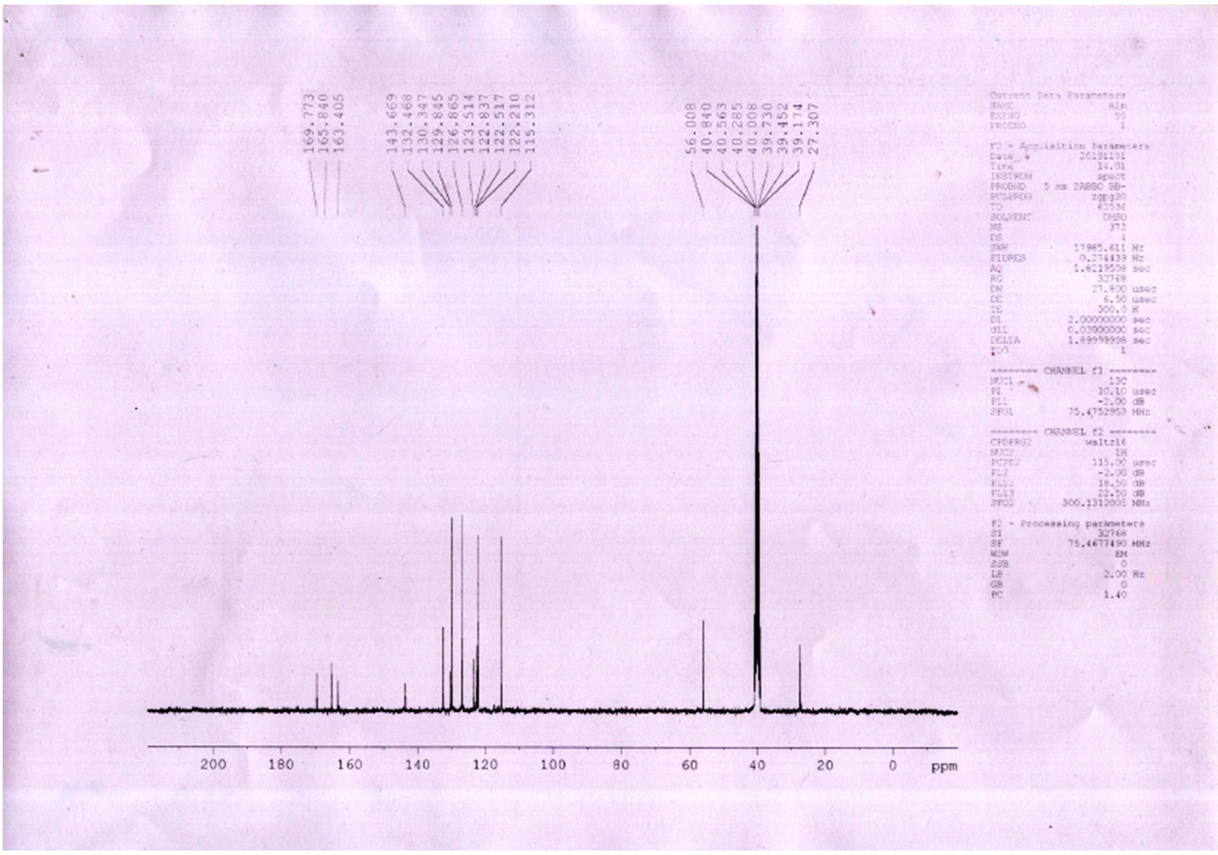

Figure S17:^{13}C NMR of compound 13

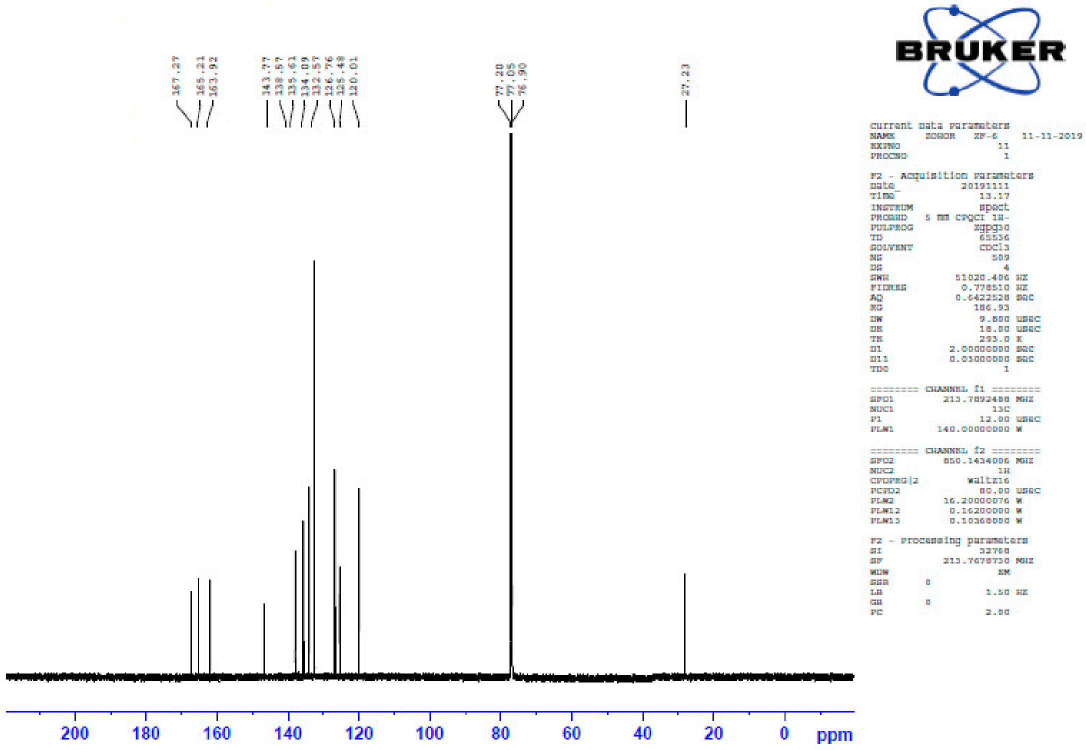

Figure S18:^{13}C NMR of compound 14

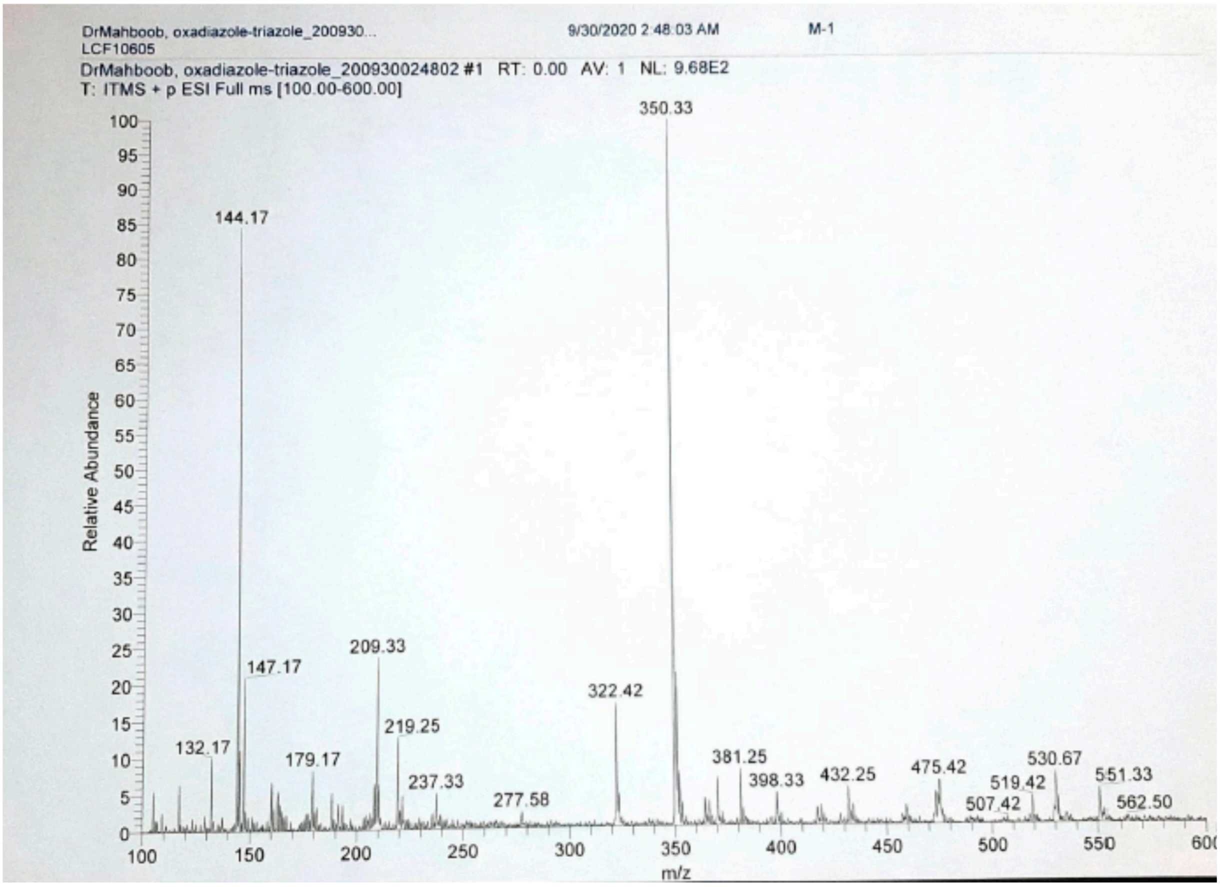

Figure S19:Mass of compound 6

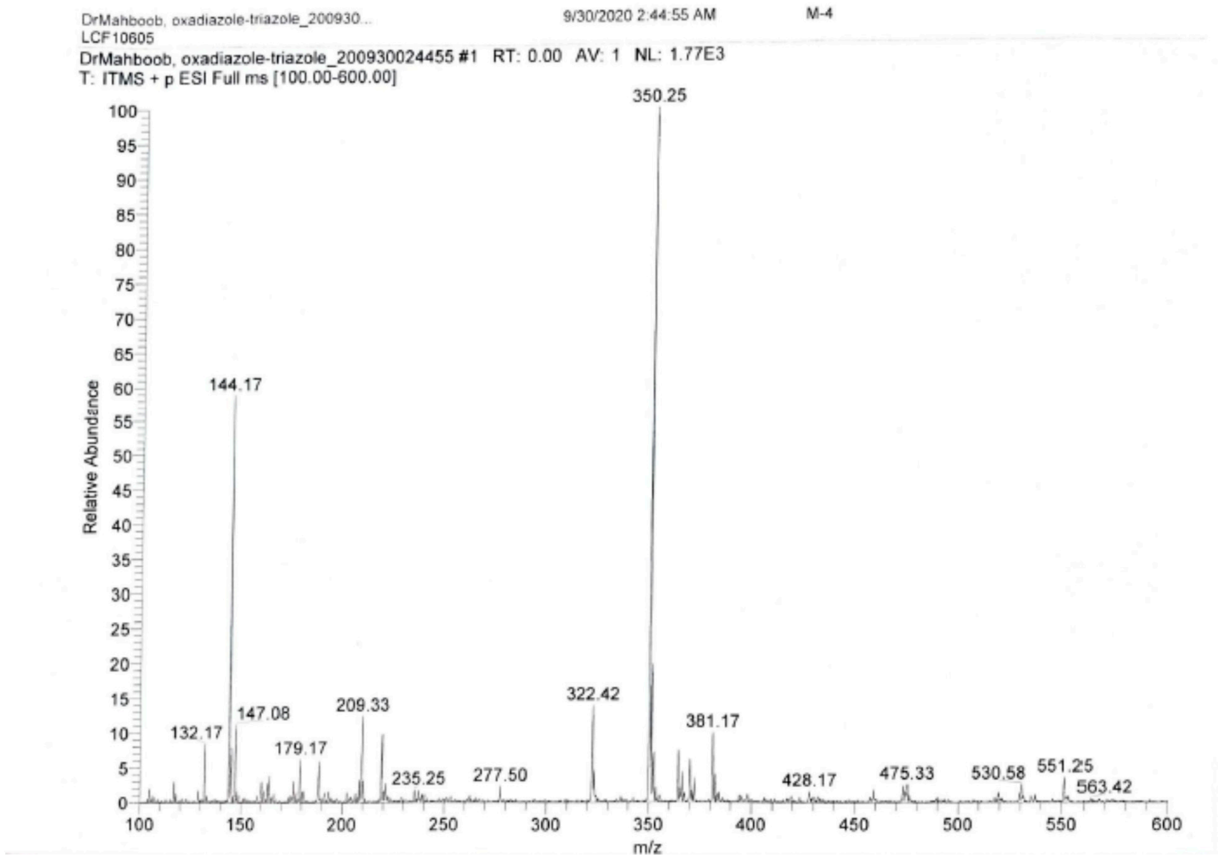

Figure S20: Mass of compound 7

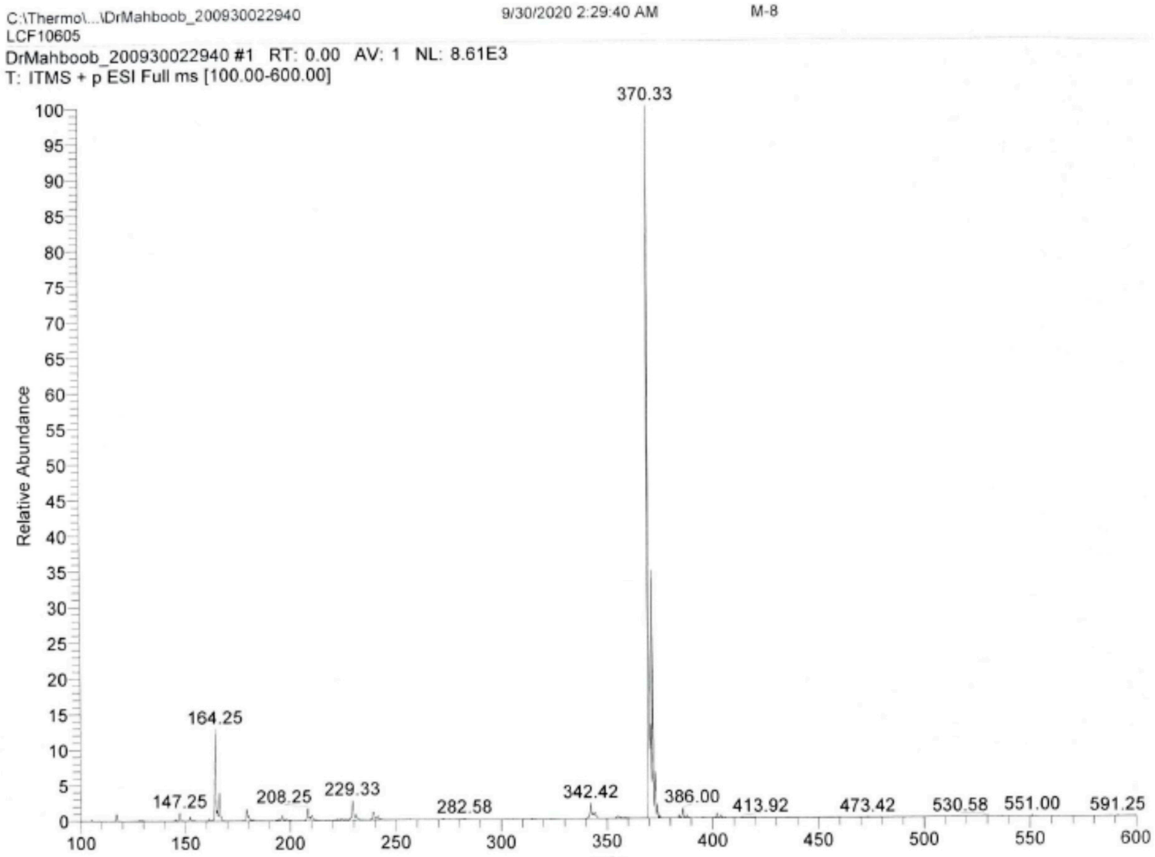

Figure S21:Mass of compound 8

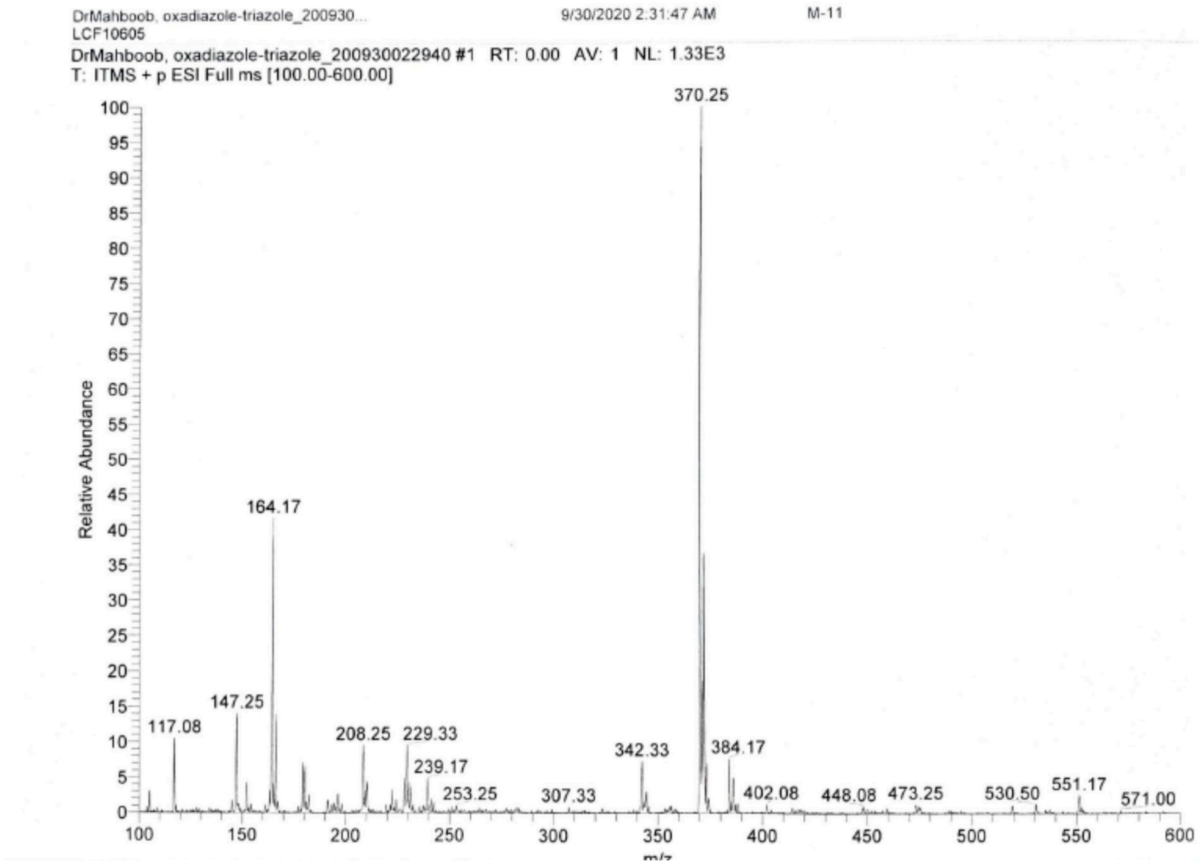

Figure S22:Mass of compound 9

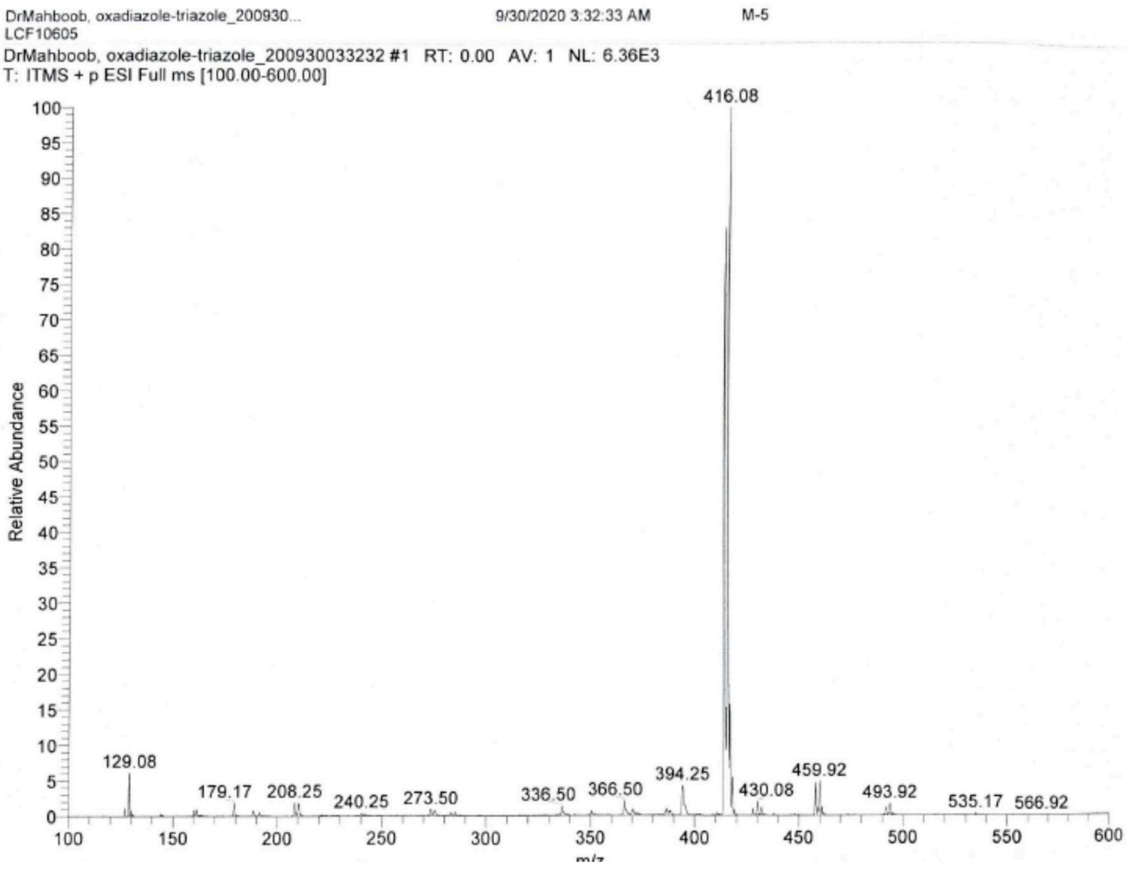

Figure S23:Mass of compound 10

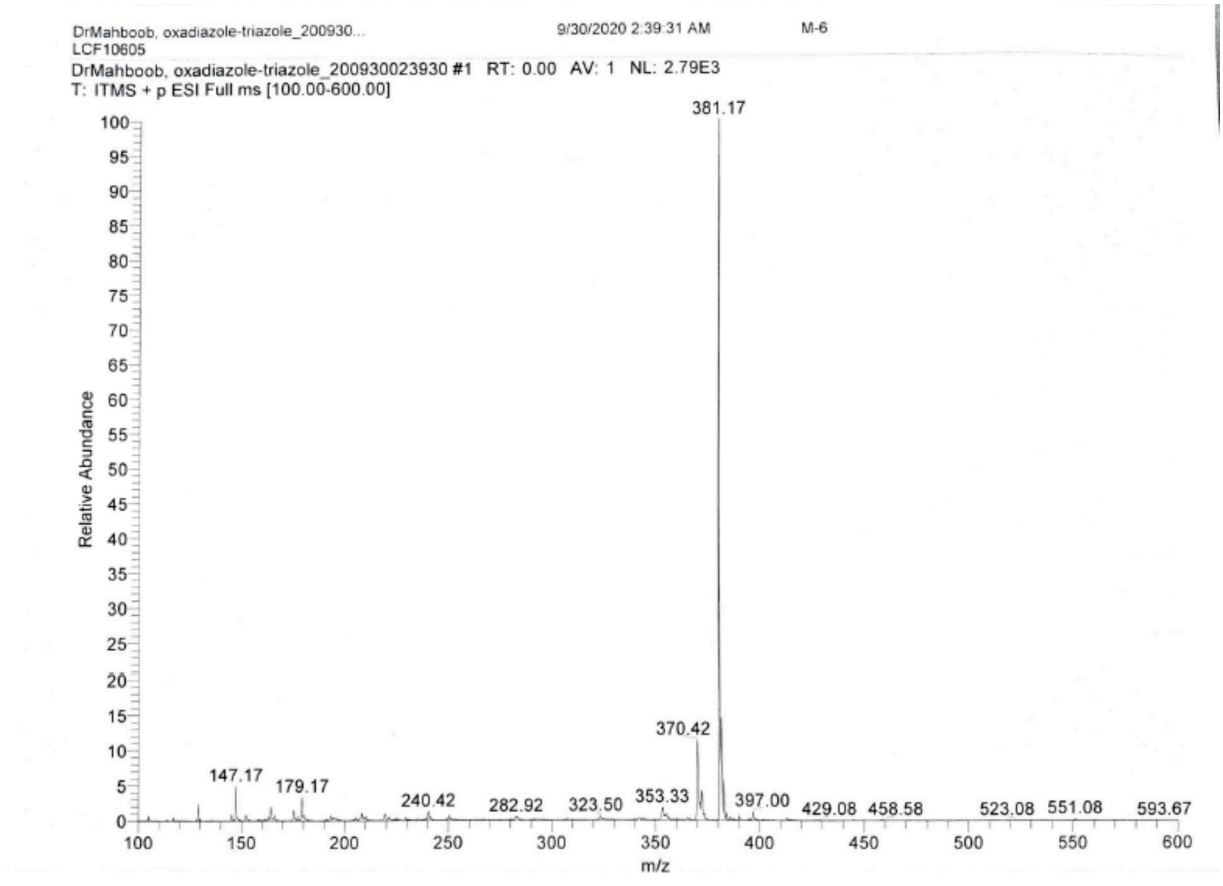

Figure S24:Mass of compound 11

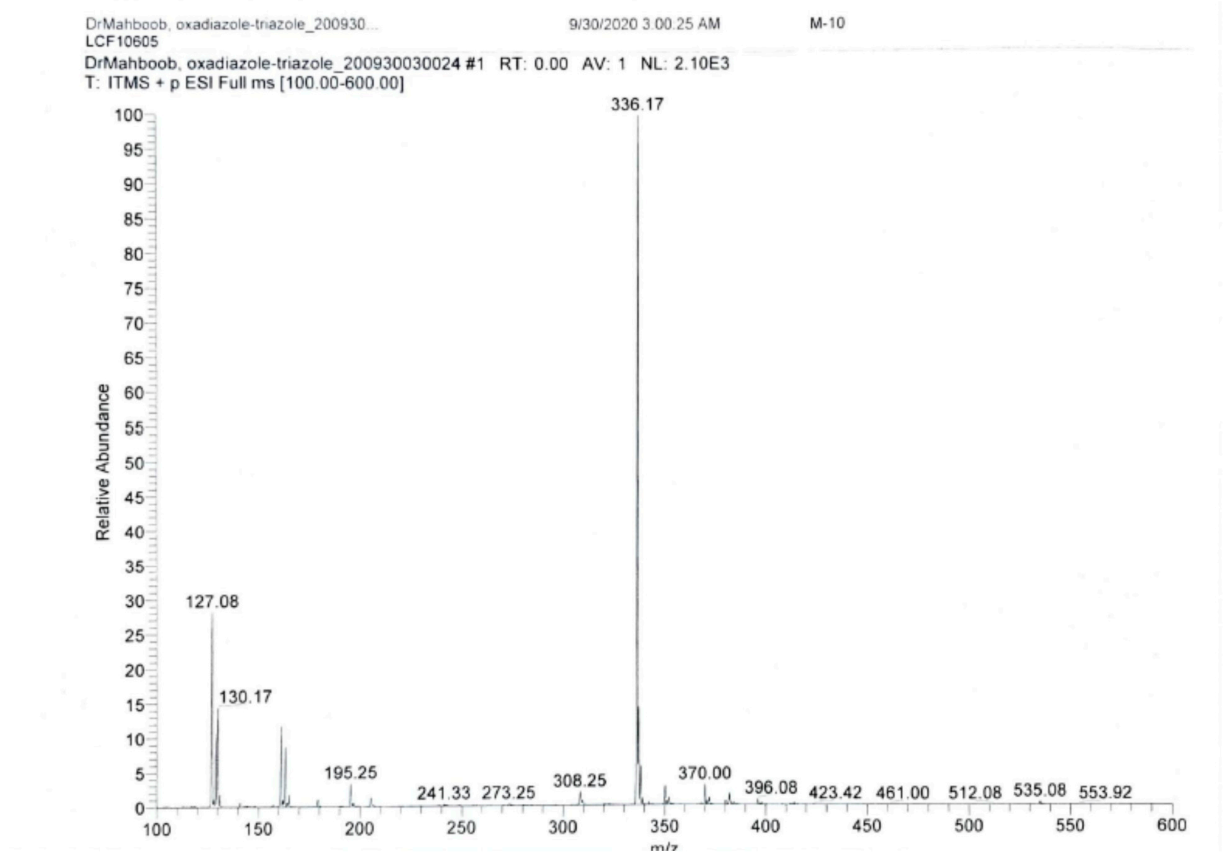

Figure S25:Mass of compound 12

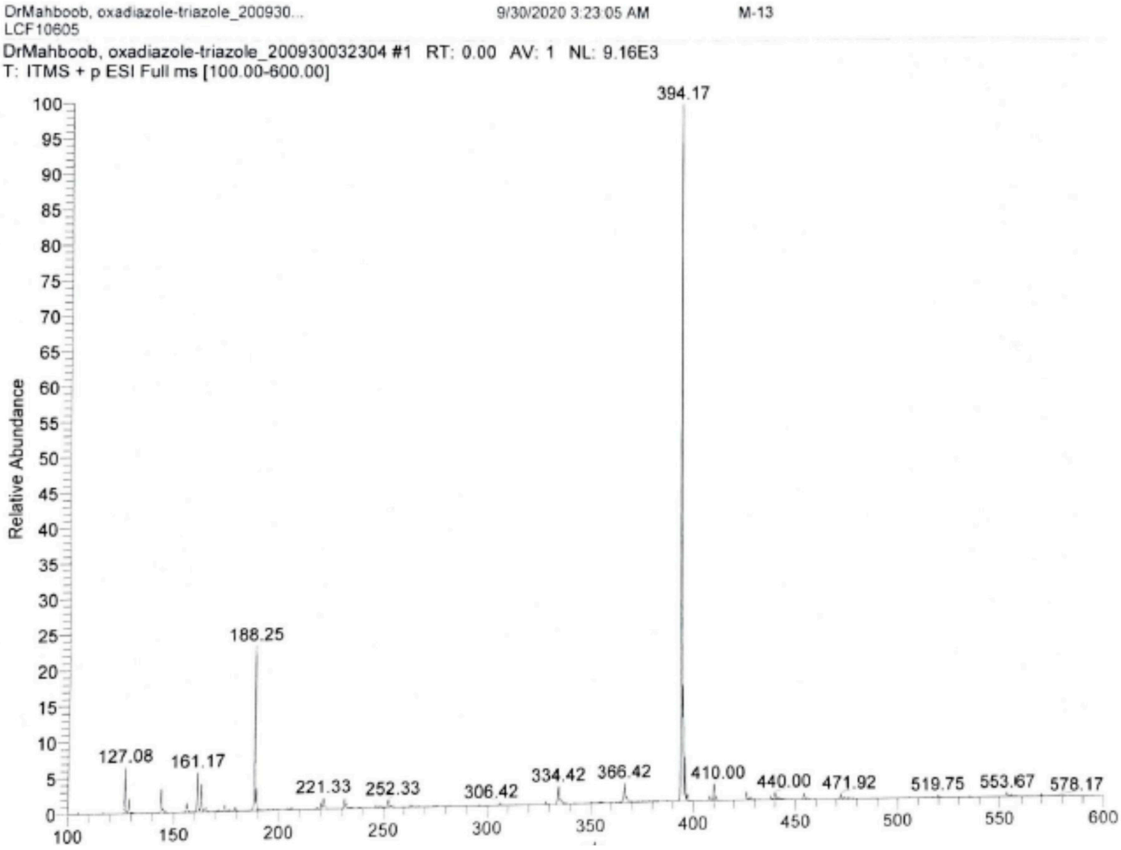

Figure S26:Mass of compound 13

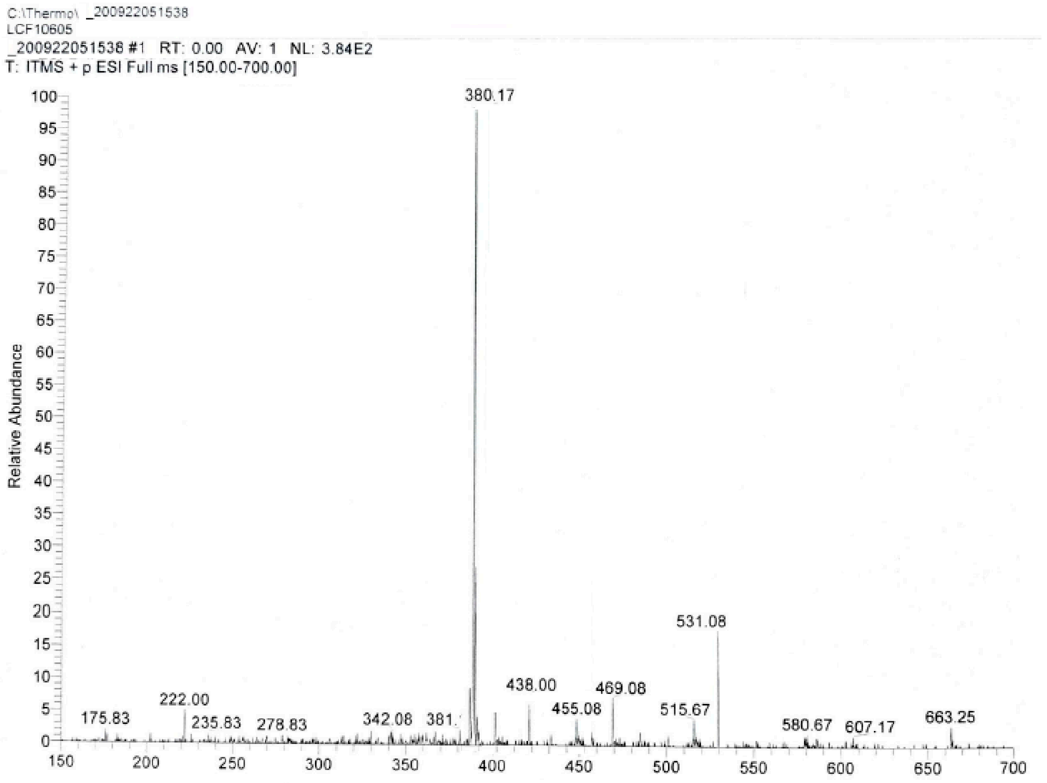

Figure S27:Mass of compound 14
